# Using multiple computer-predicted structures as molecular replacement models: application to the antiviral mini-protein LCB2

**DOI:** 10.1107/S2052252525005123

**Published:** 2025-06-23

**Authors:** Svetlana A. Korban, Oleg Mikhailovskii, Vladislav V. Gurzhiy, Ivan S. Podkorytov, Nikolai R. Skrynnikov

**Affiliations:** a Laboratory of Biomolecular NMR, St Petersburg State University, St Petersburg, 199034, Russian Federation; b NRC Kurchatov Institute – PNPI Gatchina 188300 Russian Federation; c Utrecht, The Netherlands; d Crystallography Department, Institute of Earth Sciences, St Petersburg State University, St Petersburg, 199034, Russian Federation; e Department of Chemistry Purdue University West Lafayette IN 47907 USA; Chinese Academy of Sciences, China

**Keywords:** LCB2, molecular replacement, protein crystallography, computer-predicted structures, rotamers, side-chain conformations, multiconformer ensembles

## Abstract

In this report, we describe a set of structures of the engineered protein LCB2 that has been solved starting from different computer-predicted molecular replacement (MR) models. We found that *AlphaFold3*, *AlphaFold2*, *MultiFOLD*, *Rosetta*, *RoseTTAFold* and *trRosetta* all produced successful MR models for this three-helix bundle 58-residue protein, while some of the older predictors failed. To assign *B* factors in the MR models we used the predictor-generated confidence scores or, as a convenient alternative, the accessible surface area (ASA) values. The process of multi-start structure determination using *Coot* and *Phenix* demonstrated good convergence, leading to six structures within 0.25 Å (all-atom RMSD) of each other. Of note, structural differences between the computer-predicted MR models and the final structures can be largely attributed to a single specific crystal contact. Comparing the six structural solutions, we observe that a number of surface side chains have been solved with different conformations. Interestingly, for each individual structure the electron density is consistent with a single rotameric state and offers no direct evidence of conformational heterogeneity. Strictly speaking, this behavior constitutes a case of model bias; we argue, however, that it represents a benign side of model bias. Specifically, when we use a model where the side-chain conformation corresponds to one of the actual (significantly populated) rotameric states, this leads to an enhancement of the electron density for this particular conformation. Conversely, when we use a model with an irrelevant (low-population) side-chain conformation, it fails to produce the matching electron density. We thus conclude that the six LCB2 structures obtained in this study can be grouped into a multiconformer ensemble, where structural variations are representative of protein’s conformational dynamics. Indeed, using this six-member ensemble leads to a significant drop in *R*_work_ and *R*_free_ compared with the individual solutions. This interpretation was also supported by our MD simulations of the LCB2 crystal.

## Abstract 2

A set of crystallographic structures has been obtained for the small antiviral protein LCB2 using molecular replacement models from six different structure-prediction programs. This set of structures can be interpreted as a multiconformer ensemble, improving quality metrics and offering an interesting insight into side-chain dynamics.

## 1. Introduction

At the present time, 74% of all crystallographic protein structures in the Protein Data Bank (PDB) have been solved using the molecular replacement (MR) technique. Traditionally, MR models have been obtained using crystallographic structures of homologous proteins. It has been customary to edit such homology models. The editing may involve partial or complete removal of side chains, as well as removal of loops that have low sequence similarity to the target or appear conformationally disordered (as deduced from consideration of multiple search models) (Evans & McCoy, 2008 ▸; Abergel, 2013 ▸). More sophisticated search models have also been employed, such as ensemble models (Kleywegt & Jones, 1996 ▸) or models adjusted to account for domain dynamics in multidomain proteins (Suhre & Sanejouand, 2004 ▸).

Over time, crystallographers started using specialized modeling programs to improve on homology templates. The first such example in the PDB was 1qnw (Loris *et al.*, 2000 ▸), which was solved with the help of the homology modeling tool *SWISS-MODEL* (Guex & Peitsch, 1997 ▸). Soon thereafter the structure 1qym was reported (Manjasetty *et al.*, 2004 ▸), where the MR model was based not on a single homology template, but rather on several templates automatically selected by *SWISS-MODEL*. At about the same time the structure 1row (Schormann *et al.*, 2004 ▸) was solved using the homology template prepared by* MODELLER* (Šali & Blundell, 1993 ▸). The option of using *MODELLER* to improve on homology templates has also been offered by the CaspR server (Claude *et al.*, 2004 ▸).

It should be noted, however, that initially the attempts to use modeling programs to improve on homology templates met with only very limited success. Special tests to investigate this matter have been conducted as a part of the biannual Critical Assessment of Structure Prediction (CASP) competition. The first such test under the ‘model refinement’ category showed that only a small fraction of all treated templates were actually improved, and even then the improvement was mostly marginal (MacCallum *et al.*, 2009 ▸). Over the next decade there was some progress in this area, but the results still left much to be desired (Read *et al.*, 2019 ▸). Because of the recent successes in *ab initio* structure predictions (see below), this CASP category has been discontinued (Millán *et al.*, 2021 ▸).

While homology-based predictors rely on the information from a limited number of homologous structures, *ab initio* predictors (also known as *de novo* predictors) make use of a generalized knowledge of the principles of protein architecture that is derived from a large body of structural data, such as the entire PDB repository. It should be noted that the boundary between homology-based and *ab initio* predictors is blurred: many predictors that have their roots in homology modeling have evolved into more general instruments utilizing large amounts of structural information. One of the early leaders in the field of *ab initio* structure prediction was *Rosetta*; over time this program has gone through a number of transformative changes (Das & Baker, 2008 ▸; Yang *et al.*, 2020 ▸; Watson *et al.*, 2023 ▸). Using *Rosetta*, Baker and co-workers first demonstrated that an *ab initio* protein model can successfully be used as an MR model (Qian *et al.*, 2007 ▸). A number of studies followed, where *ab initio* MR models were tested for dozens and even hundreds of crystallographic datasets (Rigden *et al.*, 2008 ▸; Das & Baker, 2009 ▸; Rämisch *et al.*, 2015 ▸); a special platform, *AMPLE*, was developed to support such applications (Bibby *et al.*, 2012 ▸; Keegan *et al.*, 2015 ▸; Simpkin *et al.*, 2019 ▸). New crystallographic structures solved with the help of *Rosetta*-generated *ab initio* MR models started to emerge as well (Sun *et al.*, 2018 ▸; Takekawa *et al.*, 2019 ▸).

Three years ago, the highly accurate neural-network-based predictor *AlphaFold2* took the field by storm (Jumper *et al.*, 2021 ▸). Tests have shown that *AlphaFold2* can generate MR models with a success rate of *ca* 90% (Millán *et al.*, 2021 ▸; McCoy *et al.*, 2022 ▸; Pereira *et al.*, 2021 ▸; Terwilliger *et al.*, 2023 ▸). Two very recent studies found that as many as 92% of the structures that were originally determined using single-wavelength anomalous diffraction can successfully be solved by means of the (appropriately edited) MR models by *AlphaFold2* (Keegan *et al.*, 2024 ▸; Wang *et al.*, 2025 ▸). Taking advantage of this new opportunity, a number of structures have been solved using diffraction datasets that defied previous structure-solving efforts (Kryshtafovych *et al.*, 2021 ▸; Barbarin-Bocahu & Graille, 2022 ▸). Not surprisingly, the number of structures determined with the help of MR models from *AlphaFold2* has grown dramatically in recent years. By early summer 2023, the PDB contained 224 identifiable structures that have been solved in this manner. Only a year later we found 911 such structures. As of this day the number has reached 2142 (this includes the structures solved with the help of *AlphaFold3*). The actual number is probably even higher because some depositors indicate that MR models have been generated *in silico* without providing any further details.

Other programs strive to replicate this success. In particular, *RoseTTAFold* has similar capabilities to *AlphaFold2* and also led to a number of *de novo* protein structures (Baek *et al.*, 2021 ▸). New technologies have also been incorporated into the recently released *D-I-TASSER* and *D-QUARK* servers (Zheng *et al.*, 2021 ▸; Zheng *et al.*, 2023 ▸). *ESMFold* employs an algorithm which is nearly as accurate as *AlphaFold2*, but computationally is much faster (Lin *et al.*, 2023 ▸). *MultiFOLD* and its companion programs bring to the table the ability to model protein assemblies (McGuffin *et al.*, 2023 ▸). Finally, the arrival of *AlphaFold3* sets a new benchmark in this rapidly progressing field (Abramson *et al.*, 2024 ▸). Methodological studies in the area of protein structure prediction also continue at a rapid pace (Huang *et al.*, 2023 ▸).

In this communication we take an approach that is somewhat different from most tests of computer-generated MR models. Specifically, instead of testing a single predictor across a set of crystallographic structures, we have tested a number of predictors on a single structure. The structure is that of a small (58 residues) engineered protein, LCB2, which was developed in Baker’s laboratory as a blocking ligand of the SARS-CoV-2 spike protein (Cao *et al.*, 2020 ▸). It was envisioned that solutions of mini-proteins such as LCB2 could be used in the form of a nasal spray to confer temporary protection against the coronavirus infection. Two of the higher-affinity mini-proteins, LCB1 and AHB2, saw further development, proving their effectiveness in animal models (Case *et al.*, 2021 ▸; Hunt *et al.*, 2022 ▸). While LCB2 was validated by means of biolayer interferometry experiments and tested in cell culture assays employing live virus, it has not been developed further.

We have found that the original design model of LCB2 (in complex with the receptor-binding domain of the viral spike protein) (Cao *et al.*, 2020 ▸) offers a *bona fide* MR model to solve the crystallographic structure. This is not surprising since there is a fairly long history of *Rosetta* design models being successfully used as MR models in crystallographic studies (Chen *et al.*, 2019 ▸; Boyken *et al.*, 2019 ▸). Furthermore, all-helical proteins, such as LCB2, are known to be best suited for this type of application (Bibby *et al.*, 2012 ▸). In addition, we have also obtained successful MR models from *AlphaFold3*, *AlphaFold2*, *MultiFOLD*, *RoseTTAFold* and *trRosetta* (Du *et al.*, 2021 ▸). A few other predictors did not reach the level of accuracy that is required of an MR model.

We performed the process of structure determination beginning with each of the six productive computer-predicted MR models. For each starting model the procedure was performed independently using the standard iterative protocol involving *Coot* (Emsley *et al.*, 2010 ▸) and *Phenix* (Liebschner *et al.*, 2019 ▸). While the initial models showed appreciable variability (up to 0.87 Å) and differed substantially from the final coordinates (up to 1.08 Å), the structure determination process fully converged, resulting in six refined models within 0.25 Å from each other. This kind of precision can be expected for a diffraction dataset with 2.1 Å resolution, as acquired in our study.

The idea of screening of multiple candidate MR models (search models) is well established in protein crystallography (Keegan & Winn, 2007 ▸; Stokes-Rees & Sliz, 2010 ▸). The purpose of such a screening procedure is normally to discover one usable MR model leading to the unique correct solution. In this study we adopted a somewhat different perspective. We employed six productive MR models to arrive at an ensemble of six distinct final structures, which are very similar yet not identical. It turns out that the differences are not limited to small atom displacements – some of the differences are more significant. In particular, a number of surface side chains were solved with different conformations.

Interestingly, for each of the individual structures the electron density for a given side chain is consistent with a single rotameric state – and offers no evidence (or little evidence) of conformational heterogeneity. However, when we compare the structures obtained from different MR models, we observe the presence of multiple side-chain conformations. Strictly speaking, this is an example of model bias whereby the final solution is dependent on a prior model. The biased character of the solutions (in terms of the side-chain conformations for several specific residues) was confirmed by inspection of the OMIT maps (Liebschner *et al.*, 2017 ▸).

Traditionally, model bias is considered to be a crystallographer’s enemy (Hodel *et al.*, 1992 ▸). However, here we argue that model bias can also be a crystallographer’s friend. Specifically, when we use a model where the side-chain conformation corresponds to one of the rotameric states that is significantly populated in the actual crystal, we thereby ‘tease out’ the electron density for this particular conformation. When we use several relevant models, we therefore recover the conformational ensemble of the side chain in question. Indeed, treating the collection of six LCB2 structures as a multiconformer ensemble, we observe a significant drop in *R*_work_ and *R*_free_ values compared with the individual structures. The conformational disorder at the sites of interest was also confirmed by a series of crystal MD simulations.

## 2. Materials and methods

Details of LCB2 expression and purification, functional assay using isothermal titration calorimetry, protein crystallization, diffraction data collection and processing, principal component analyses and MD modeling are provided as a part of the supporting information.

### 2.1. MR models

*AlphaFold3*. The web server https://www.alphafoldserver.com was used to generate *ab initio* models of LCB2 (web addresses of structure-prediction servers employed in this study are summarized in Table S3). Five models were manufactured, which is the default output for this server. The protein sequence was the same as employed in our crystallographic study (see the supporting information). Note that *AlphaFold3* was trained on the PDB structures released before 30 September 2021 and therefore has not seen our crystallographic structure 8c3e. The models, which do not include hydrogen atoms, contain predicted local distance difference test (pLDDT) scores (Mariani *et al.*, 2013 ▸) placed in the field that is normally reserved for *B* factors. To further process the models, we have used three different protocols:

(i) Conversion of pLDDT scores to *B* factors using *phenix.process_predicted_model* (Oeffner *et al.*, 2022 ▸); in doing so, several atoms with lower confidence scores, pLDDT < 70, were deleted according to the default setting ‘remove low-confidence residues’: Ser2 O^γ^, Lys29 N^ζ^, Arg41 N^η1^ and Arg41 N^η2^, Arg49 N^η1^ and Arg49 N^η2^, Arg52 N^η1^ and Lys56 N^ζ^.

(ii) Generation of *B* factors based on the degree of solvent exposure of the individual atoms in the model using *phenix.sculptor* (Bunkóczi & Read, 2011*b* ▸).

(iii) Assignment of the same constant value to all atomic *B* factors using *phenix.sculptor* (*e.g.* 10 or 20, the specific value does not matter for the subsequent *phenix.phaser* treatment).

In this manner, we produced 15 starting models, which differed from each other by atomic coordinates and/or *B*-factor values. All of these models were processed by *phenix.phaser* (McCoy *et al.*, 2007 ▸). In doing so, we indicated that the expected number of protein molecules in the asymmetric unit was 2 (although in reality there is only 1 protein molecule per asymmetric unit). This proved to be a helpful initial guess because it effectively accounts for the presence of *ca* 50% twinning in our crystal and thus provides the program with a better initial estimate of the σ_*A*_ function (McCoy, 2017 ▸). Starting with this estimate, *phenix.phaser* consistently arrived at superior solutions characterized by higher log-likelihood gain (LLG) scores and translation function Z-scores (TFZ scores). Appropriately, these solutions featured a single protein molecule in the asymmetric unit. The complete list of 15 *AlphaFold3*-derived models with their respective LLG and TFZ scores is given in Table S4. From this list we selected the model with the highest scores, see Table 1 ▸, and used it as a starting point to solve the structure via the standard *Phenix*- and *Coot*-based procedure.

*AlphaFold2*. *AlphaFold2* version 2.3.2 was accessed through the official Colab Notebook, see Table S3. A single *ab initio* model of LCB2 was generated, which is the default output for this server. The model originally included hydrogen atoms, which were deleted prior to *phenix.phaser* treatment. Using three different protocols to assign *B* factors, we arrived at three MR models containing residues 1 to 58 and a full complement of atoms. The best of these models, see Table 1 ▸, was used to solve the structure of LCB2. In this particular case, the process of the structure determination was performed twice, resulting in a pair of very similar, but not identical, structures.

*MultiFOLD*. Five *ab initio* models of LCB2 were generated using the designated web server, see Table S3. The models originally included hydrogen atoms, which were deleted prior to *phenix.phaser* treatment. When processing the models with *phenix.process_predicted_model*, two N-terminal residues (Gly–Ser) were deleted according to the default setting ‘remove low-confidence residues’.

*Rosetta*. The *Rosetta* model was from the archive included in the supporting information of the paper by Cao *et al.* (2020 ▸). The model is a complex of the receptor-binding domain of the SARS-CoV-2 spike protein with LCB2 (whose original sequence does not include the N-terminal Gly–Ser residues). All *B* factors were originally assigned zero values. Prior to processing with *phenix.phaser*, the spike protein was removed from the model and the hydrogen atoms were deleted. Since pLDDT scores were not available for this particular Rosetta model, the *B* factors were either calculated based on accessible surface, option (ii), or all assigned the same constant value, option (iii). The better of the two resulting models was chosen for the role of the MR model.

*RoseTTAFold*. Five *ab initio* models of LCB2 were generated using the designated web server, see Table S3. The models included estimated uncertainties (root-mean-square deviations) of the atomic coordinates, calculated internally by *RoseTTAFold* based on pLDDT scores (Oeffner *et al.*, 2022 ▸). These values were converted to *B* factors using the relevant option in the program *phenix.process_predicted_model*. The same program also deleted four N-terminal residues (Gly–Ser–Ser–Asp) and one C-terminal residue (Leu), which were classified as low-confidence regions, RMSD > 1.5 Å. Out of 15 distinct models with different atomic coordinates and/or *B* factors, the best one was used for *Coot*- and *Phenix*-assisted structure determination.

*trRosetta*. Five *ab initio* models of LCB2 were generated using the designated web server, see Table S3. The models were missing all arginine N^η1^ and N^η2^ atoms as well as tyrosine O^η^ atoms, and were used as such without any attempts to rebuild these atoms. *B* factors were assigned using options (i), (ii) and (iii), resulting in 15 distinct models, of which one model with the highest LLG and TFZ scores was chosen for further structure determination.

Similar schemes were also used for the *QUARK*, *Phyre2*, *I-TASSER* and *SWISS-MODEL* predictors. None of these methods led to productive MR models; therefore, we do not provide any further details on these efforts. The more recently released versions *D-I-TASSER* and *D-QUARK* were unsuit­able for the purpose of this study because these programs are familiar with our crystallographic structure 8c3e. We also tested the recent predictor *ESMFold*, but found that its predicted model was very similar to the one generated by *MultiFOLD*; therefore we did not include it in subsequent analyses.

The refinement statistics shown in this paper are from *phenix.refine* (Afonine *et al.*, 2012 ▸). In the case of ensemble interpretation, *phenix.refine*-reported model structure factors from the six final structures of LCB2 were averaged prior to calculation of *R*_work_ and *R*_free_. In order to compare different MR models with each other and with the final structures, we defined the set of atoms shared by all of these models (structures). This ‘consensus set’ includes residues from 5 to 57, leaving out all N^η1^ and N^η2^ arginine atoms and all O^η^ tyrosine atoms, as well as N^ζ^ atoms from residues Lys29 and Lys56. All structural superpositions were generated using the final structure derived from the *AlphaFold3* model as a reference.

## 3. Results

### 3.1. Structure overview

Table 1 ▸ summarizes the information about the computer-predicted models that were tested as potential MR models to solve the crystallographic structure of LCB2. As detailed in *Materials and methods*[Sec sec2], almost all predictors generate multiple models (typically, five models) in response to a service request involving the amino-acid sequence of a target protein. For all these models, we used three different methods of assigning atomic *B* factors: based on accessible surface area (ASA) values, based on pLDDT scores (where the atoms with particularly poor scores were removed) or using a single constant value. All of the obtained variants were assessed by means of *phenix.phaser*, and one best model for each predictor was selected for further analyses. As usual, we have used LLG and TFZ scores as quality metrics (Read & McCoy, 2016 ▸; Bunkóczi *et al.*, 2013 ▸). A complete summary of the *phenix.phaser* trials is given in Table S4, whereas the best models for all predictors are listed in Table 1 ▸.

One lesson that we learned from this exercise is that ASA-based assignment of *B* factors and pLDDT-based assignment of *B* factors lead to comparable results, whereas using uniform *B* factors is clearly less efficient (see Table S4). This means that pLDDT scores are not necessary to prepare a state-of-the-art MR model, since the long-known ASA-based approach provides a convenient alternative.

Inspection of LLG and TFZ scores in Table 1 ▸ immediately suggests that six predictors have produced successful MR models: *AlphaFold3*, *AlphaFold2*, *MultiFOLD*, *Rosetta*, *RoseTTAFold* and *trRosetta*. At the same time, four of the older programs, *QUARK*, *Phyre2*, *I-TASSER* and *SWISS-MODEL*, apparently fell short (with a typical TFZ score of 5, these models are unlikely to produce a viable solution). Indeed, our efforts to solve a structure were successful with the former six MR models, but unsuccessful with the latter four.

In addition to the single best *AlphaFold3* model (LLG = 213, TFZ = 16), we also considered an ensemble comprised of five *AlphaFold3* models. In our tests using *phenix.ensembler* and *phenix.phaser*, this ensemble produced somewhat improved scores (LLG = 227, TFZ = 17). Apparently, the set of models generated by *AlphaFold3* to some degree captures the conformational variability of LCB2 (Bunkóczi & Read, 2011*a* ▸). One may expect that in certain borderline situations use of the computer-predicted ensembles (subject to trimming as needed) may tip the scales, leading to successful structure determination.

Turning to the usage statistics for computer-predicted MR models (the fourth column in Table 1 ▸), we observe that *AlphaFold2* followed by *AlphaFold3* have enjoyed great success as sources of workable MR models. Our results suggest, however, that other predictors, both preceding *AlphaFold2* (*e.g.Rosetta*) and building on *AlphaFold2* technology (*e.g.MultiFOLD*), can also produce *bona fide* MR models. This situation creates an opportunity for comparative analyses of LCB2 structures derived from different computer-predicted MR models.

Listed in Table 2 ▸ are the refinement statistics for LCB2 structures obtained from the MR models by six different predictors. The structures are characterized by *R*_work_ of 0.21 and *R*_free_ of 0.24–0.25, typical of a crystallographic resolution of 2.10 Å. Local geometry measures, such as Ramachandran and rotamer outliers, clash scores and other metrics that are conveniently summarized in the *MolProbity* scores (Williams *et al.*, 2018 ▸), are very good, reflecting the high quality of the automated refinement using *phenix.refine* (Afonine *et al.*, 2012 ▸). All structures include residues from 4 to 58, with no alternative conformations; they also include from 1 to 4 glycerol molecules and between 18 and 24 water molecules. Based on these standard statistics it is hardly possible to rank the structures and identify the best (most accurate) set of coordinates. Instead, we argue that all of the structures are equally valid and one cannot be chosen over the other (as discussed further in this paper).

**Figure 1:**
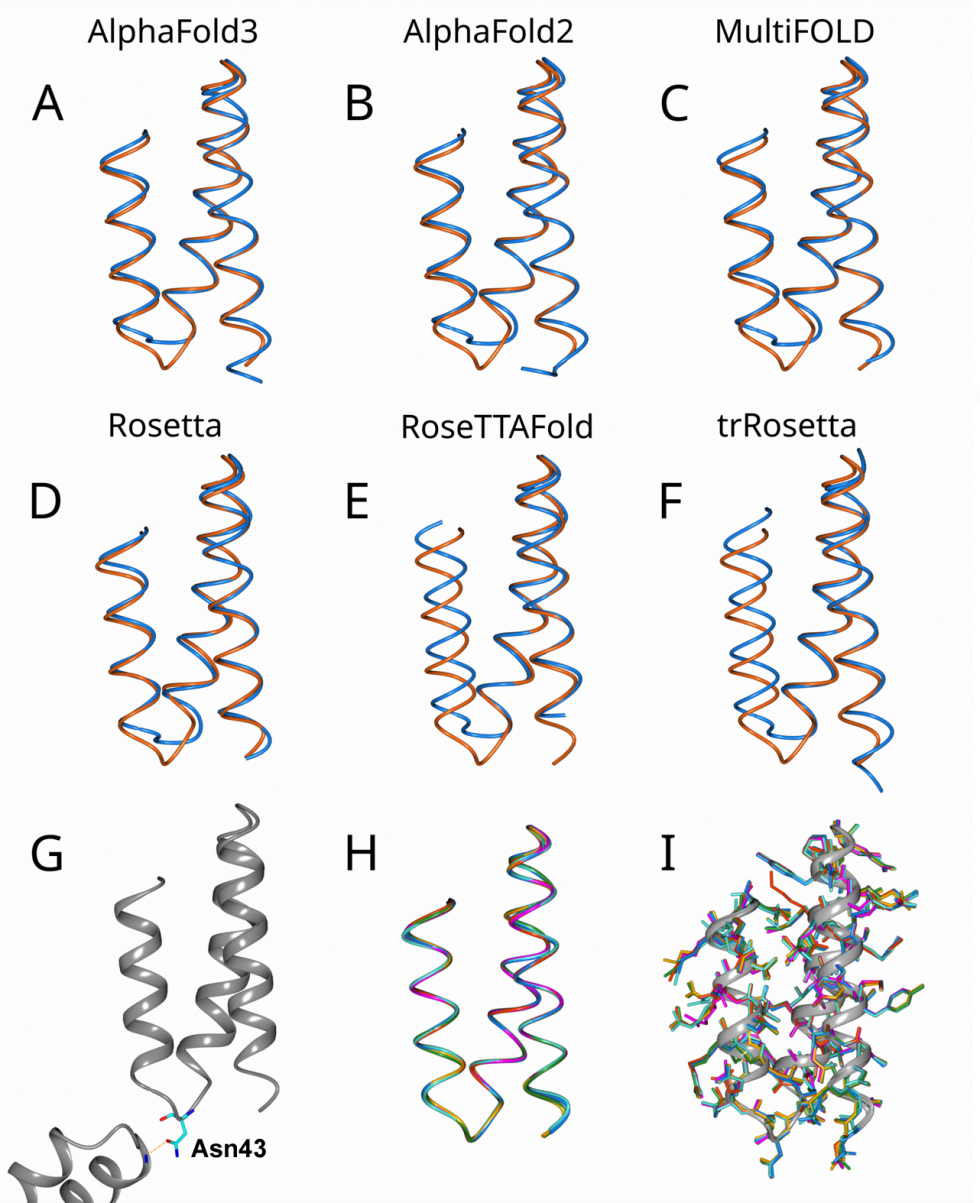
MR models and their corresponding structures for the small antiviral protein LCB2. (*a*)–(*f*) Superposition of the final structures (red) and their parent MR models (blue, annotated at the top of the panels). The structures are superimposed via C^α^ atoms from the first and second helices (residues 6–22 and 25–41). (*g*) Intermolecular hydrogen bond between residues Asn43 and Glu46 in the structure derived from the *AlphaFold3* model (for visual clarity, we do not show the symmetry-equivalent hydrogen bond). (*h*) Superposition of the six crystallographic structures colored according to their parent MR models: blue (*AlphaFold3*), magenta (*AlphaFold2*), green (*MultiFOLD*), turquoise (*Rosetta*), red (*RoseTTAFold*) and orange (*trRosetta*). The structures are superimposed via C^α^ atoms from the consensus set. (*i*) Superposition of the six crystallographic structures with side chains colored according to their parent MR models. The structures are superimposed via C^α^ atoms from the consensus set, as in panel (*h*). The figures were prepared using the program *CCP4MG* (McNicholas *et al.*, 2011 ▸).

Shown in Figs. 1 ▸(*a*)–1(*f*) are pairwise superpositions of the initial computer-predicted MR models and their descendant crystallographic structures. In all of these pairs we observe distinct differences in the conformation of the short loop connecting the second and third helices (residues 42–44). In addition, we observe that in the *RoseTTAFold* and *trRosetta* model the third helix is significantly shifted from its true (*i.e.* experimentally determined) position. The shift amounts to *ca* 2 Å in the case of the *RoseTTAFold* model and a little less than that for the *trRosetta* model, see Fig. 1 ▸(*e*) and Fig. 1 ▸(*f*).

The situation with the loop 42–44 led us to suggest that its conformation in the crystallographic structure is influenced by a crystal contact. Indeed, it turns out that residue Asn43 at the center of the loop forms an intermolecular side-chain-to-backbone hydrogen bond with the amide group of residue Glu46 in the symmetry-related protein molecule. This hydrogen bond apparently leads to stretching out of the loop (Rapp & Pollack, 2005 ▸), thus causing the difference between the computer-generated model and the actual structure, see Fig. 1 ▸(*g*). Apparently, we see an example of the situation where both the computer-predicted MR model and its related crystallographic structure are correct – yet they differ from each other because computer-prediction algorithms are agnostic of crystal contacts, whereas the experimental structures are (to some degree) sensitive to crystal contacts.

While the MR models show some differences from the final structures, the latter are characterized by near-identical backbone folds. The bundle in Fig. 1 ▸(*h*) shows a superposition of six structures colored according to the MR models that were used to solve these structures. All structures superimpose almost perfectly, with slight deviations observed only at the N-terminal residues 4–5. This picture indicates that the process of structure determination is well converged and, in a global sense, the final structures are free from any significant model bias.

Although the six final structures are nearly identical with respect to their backbone folds, the side chains are not as well defined. Shown in Fig. 1 ▸(*i*) is the bundle of six superimposed final structures, where the focus is on the side chains. While the majority of side chains are nicely reproduced across the entire set of structures, some others have been solved in different conformations. The origins of these differences are discussed later in this paper.

**Figure 2:**
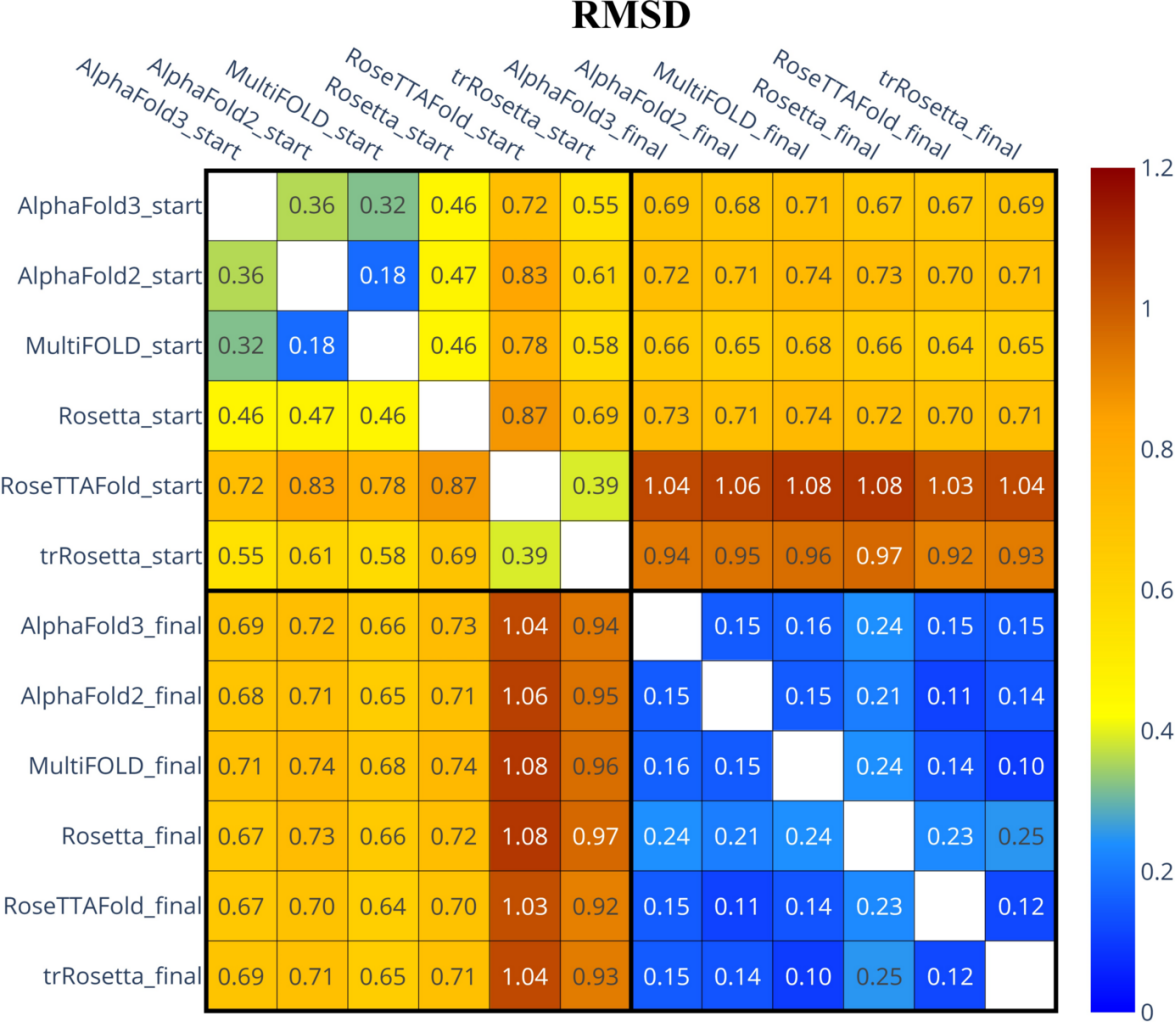
Pairwise RMSD for six computer-predicted MR models and six respective fully refined structures of LCB2. The RMSD values were calculated for all non-hydrogen atoms in the ‘consensus set’ (shared by all models/structures, see *Materials and methods*[Sec sec2]).

The differences between the initial MR models and the final structures can be conveniently quantified via pairwise root-mean-square deviation (RMSD) of the atomic coordinates. The RMSD values for all non-hydrogen atoms are summarized in the form of a heat map in Fig. 2 ▸. Considering the initial models (upper left quadrant in Fig. 2 ▸), we observe that some of the models are quite similar to each other, *e.g.* the *AlphaFold2* and *MultiFOLD* models are within 0.18 Å of each other, which reflects the genetic connection between the two programs. On the other hand, the *RoseTTAFold* and *trRosetta* models form a sort of cluster, which is appreciably different from the remaining four models, with RMSD values up to 0.87 Å. This is obviously related to the shift in the relative position of the helices, as visualized in Figs. 1 ▸(*a*)–1(*f*).

At the same time, the MR models are visibly different from the final structures (*cf*. the upper right quadrant or, equivalently, lower left quadrant in Fig. 2 ▸). For the *AlphaFold3*, *AlphaFold2*, *MultiFOLD* and *Rosetta* models, this difference amounts to *ca* 0.7 Å. This deviation is in part attributable to the conformation of the loop 42–44, see Figs. 1 ▸(*a*)–1(*f*). For the *trRosetta* and especially *RoseTTAFold* models the deviations are larger, up to 1.08 Å, due to the shift in the relative positions of the α-helices. In general terms, however, deviations on the scale of 1.08 Å or less are considered to be modest and correspond to high-quality MR models (Oeffner *et al.*, 2013 ▸). This is consistent with our initial assessment of the MR models using LLG and TFZ scores and is ultimately confirmed by the success of the structure determination procedure.

Importantly, all of the final structures are nearly identical, with all-atom RMSDs in the range 0.10–0.25 Å (lower right quadrant in Fig. 2 ▸). These numbers provide a measure of uncertainty for the LCB2 coordinates, *i.e.* characterize the precision of the structure obtained here at 2.1 Å resolution. How does this precision compare with what is reported in the literature or found in the PDB? Historically, there has been some conflicting evidence regarding the convergence of X-ray structure determination and precision of crystallographic protein structures (Daopin *et al.*, 1994 ▸; Ohlendorf, 1994 ▸). However, more recently it has been shown that structures of globular proteins can indeed be solved to a very high precision (Liebschner *et al.*, 2013 ▸).

As one relevant example, consider three structures of the well known model protein ubiquitin, 1ubq (Vijay-Kumar *et al.*, 1987 ▸), 1ubi (Ramage *et al.*, 1994 ▸) and 4xof (Ma *et al.*, 2015 ▸). The structures were solved independently in the space group *P*2_1_2_1_2_1_ with crystallographic resolutions of 1.80, 1.80 and 1.15 Å, respectively. Considering the well-structured body of ubiquitin, residues 2–72, we find that pairwise all-atom RMSD values for these three structures fall in the range 0.11–0.20 Å. This is similar to what we found for our LCB2 structures. This is also in line with various empirical estimates of structure accuracy, such as the Luzzati plot (Luzzati, 1952 ▸), the σ_*A*_ plot (Read, 1986 ▸), Cruickshank’s DPI (Cruickshank, 1999 ▸) and others, which assume that crystallographic structures are affected by random errors obeying a normal distribution. As a cautionary note, the presence of divalent ions in the same orthorhombic ubiquitin crystals as discussed above immediately increases the RMSD values to a level of *ca* 0.5 Å (Bang *et al.*, 2005 ▸; Ma *et al.*, 2015 ▸). Likewise, proteins crystallized in different space groups or with different ligands, as well as proteins carrying point mutations or post-translationally modified residues, often differ by 0.5–1.0 Å or more (Burra *et al.*, 2009 ▸). In addition, when crystallographic structures are modeled using conformational ensembles (as opposed to the traditional single-model representation), the coordinate variance within such ensembles tends to be larger than the 0.10–0.25 Å observed in this study (Zoete *et al.*, 2002 ▸; DePristo *et al.*, 2004 ▸).

In addition to RMSD, we also used two other metrics to quantify the structural differences between the MR models and their descendant structures: global distance test – high accuracy (GDT-HA) and the local distance difference test (LDDT). The former quantity provides a better measure of overall structural similarity (de-emphasizing local deviations in the loop or termini regions), while the latter focuses on local similarities (de-emphasizing more global variations that may, for instance, arise from relative positioning of domains in a multidomain protein) (Olechnovič *et al.*, 2019 ▸). The results are shown in Fig. S2, corroborating the discussion of the RMSD map above.

Finally, it is convenient to visualize the convergence of the structure determination procedure using principal component analysis (PCA) (Jolliffe, 2002 ▸). In applying this method, a special coordinate frame is employed where each of the 12 analyzed models is represented by a set of coordinates {PC1, PC2, PC3, …, PC11} such that PC1 represents the most variation and PC2 represents the second most variation in the structural data. Hence, a PC1–PC2 map puts on display the most significant differences between the structures (see the supporting information for a complete description of the method).

**Figure 3:**
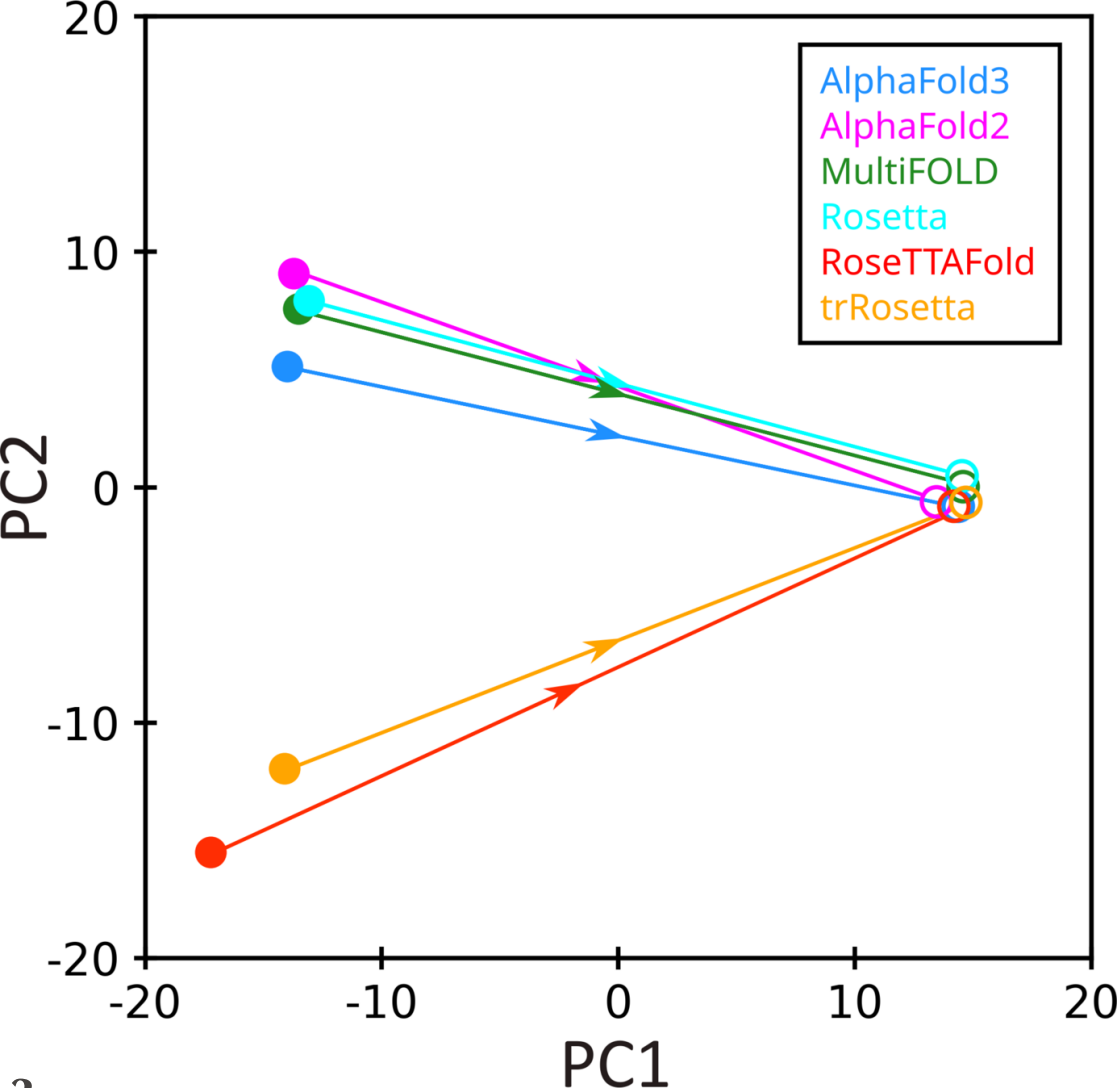
Principal component map PC1–PC2 for six MR models and six refined structures of LCB2 (solid and open circles, respectively). The structural states used in the PCA are vectors composed of the non-hydrogen atom coordinates from within the consensus region (see the supporting information for details).

Shown in Fig. 3 ▸ is a PC1–PC2 map containing six computer-generated MR models of LCB2 (solid circles) and six final structures (open circles). The identities of the models and structures are color coded as indicated in the legend, and the related models and structures are connected by arrow lines. It is immediately obvious from the plot that the MR models are sufficiently diverse and far removed from the final structures, whereas the final structures are tightly clustered, *i.e.* nearly identical. The plot nicely illustrates the convergence of the structure determination procedure as implemented in this study.

Of note, the PCA method allows one to visualize structural changes associated with PC1 and, separately, with PC2 (see the supporting information for additional information). Making use of this option, we determined that PC1 represents a mixture of the two modes: conformational change in the loop 42–44 and the positional shift of the third α-helix. Independently, the shift of the third helix is also parameterized by PC2. We further found that the combination of PC1 and PC2 fully captures the backbone variations shown in Figs. 1 ▸(*a*)–1(*f*).

### 3.2. Conformational dynamics

**Figure 4:**
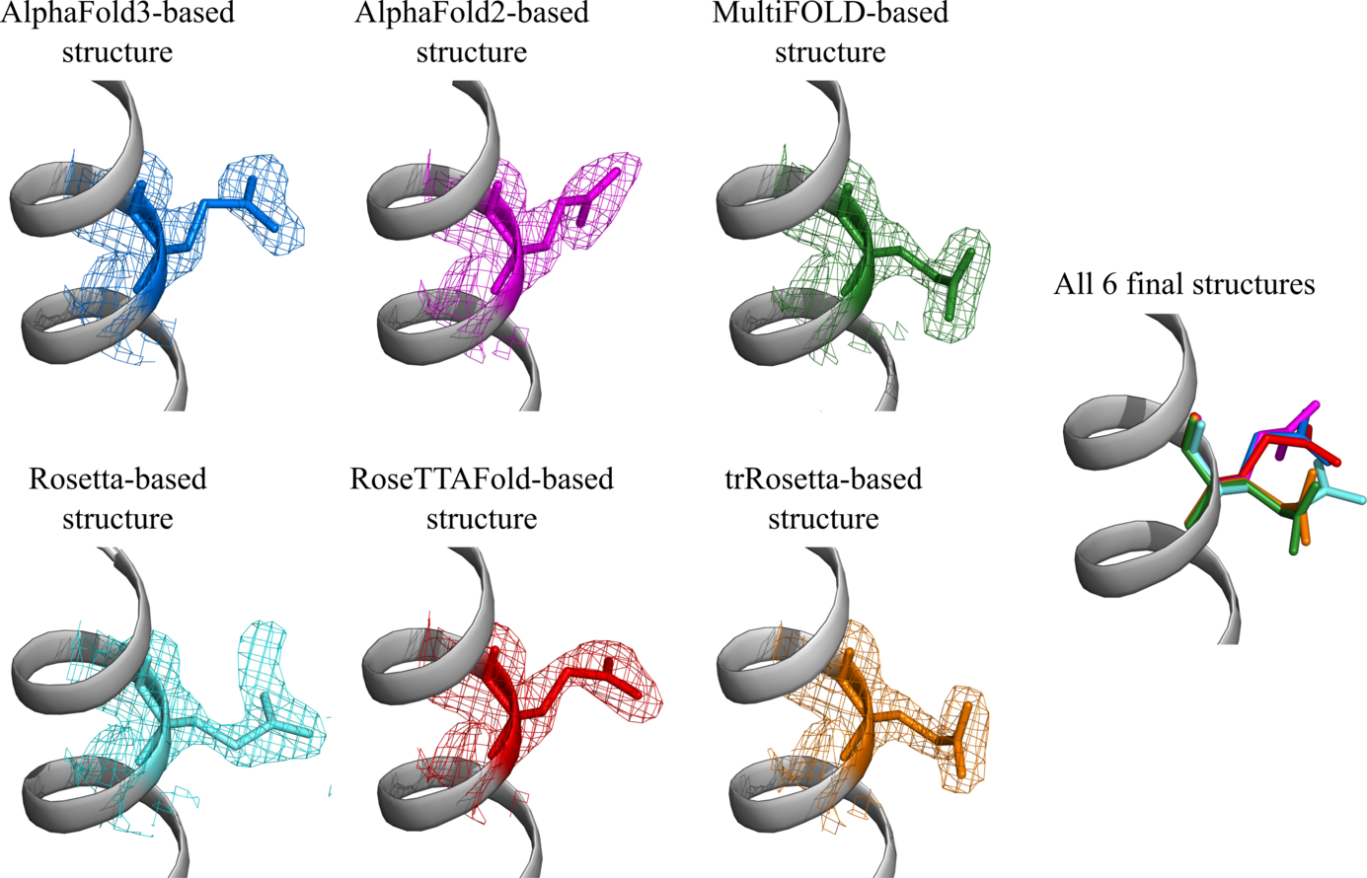
Side chain of residue Glu53 in the six final structures of LCB2. Electron density maps 2*mF*_o_ − *DF*_c_ are plotted at the level of 1σ (the differential maps *mF*_o_ − *DF*_c_ are not plotted since they show no observable density at the default level of 3σ). The color scheme is the same as in Fig. 1 ▸. In addition to Glu53, some other surface side chains in LCB2 also show conformational disorder, see Fig. S3 and Fig. S4.

While the backbone of LCB2 is almost perfectly reproduced from one solution to another, see Fig. 1 ▸(*h*), the situation with the side chains is more nuanced. While most of side chains are likewise reproduced, some of them are solved with different conformations, see Fig. 1 ▸(*i*). As one such example consider the side chain Glu53 illustrated in Fig. 4 ▸. In the structures derived from the *AlphaFold3*, *AlphaFold2* and *RoseTTAFold* models, this side chain adopts the conformational state tt 0° according to the classification by Lovell and co-workers (Lovell *et al.*, 2000 ▸). This particular glutamate conformation is rather common in the PDB (specifically, in α-helices its estimated frequency of occurrence is 25%); in the structures at hand it is additionally stabilized by ionic interaction with the nearby Arg57 side chain. In contrast, in the structures derived from the *MultiFOLD* and *trRosetta* models, the Glu53 side chain is found in a different conformational state, mm-40° (frequency of occurrence in α-helices 19%), which is stabilized by apparent ionic interactions to Arg52 and Lys56. Finally, the structure descendent from the *Rosetta* model features Glu53 in yet another conformational state, mt-10° (frequency of occurrence in α-helices 36%).

Let us summarize some observations regarding the Glu53 side chain as shown in Fig. 4 ▸. The weak electron density and elevated *B* factors suggest that the Glu53 side chain undergoes conformational dynamics. For this side chain our structure calculations lead to three different rotameric states, all of them clearly visible in the 2*mF*_o_ − *DF*_c_ map plotted at the 1σ level. In all cases but one (the *Rosetta*-based structure) the electron density does not offer any evidence of alternative conformations. Our attempts to model the Glu53 side chain using alternative conformations were unconvincing.

Strictly speaking, the results shown in Fig. 4 ▸ constitute an example of model bias, *i.e.* the situation where the final solution depends on a prior model. To further explore this point, we compiled a set of OMIT maps focused on the Glu53 residue. Specifically, we took the six refined LCB2 structures, deleted their Glu53 side chains, and used the resulting constructs to generate electron density maps. It turned out that these OMIT maps showed essentially no observable density for the Glu53 side chain, thus confirming the notion of model bias (see Fig. S5).

Traditionally, the effect of model bias in protein crystallography is viewed in a purely negative light, as a potential source of structural error. In this particular case, however, we argue that model bias can be seen from a different angle. We suggest that the Glu53 side chain is conformationally labile with several substantially populated conformational states. Assume for a moment that we have obtained a (highly refined) LCB2 model where the Glu53 side chain happens to reproduce one of these populated states. Using such a (faithful) model allows us to ‘tease out’ the electron density corresponding to this particular populated rotamer. Therefore, we argue that the results in Fig. 4 ▸ represent the actual Glu53 rotamers that are significantly populated in the LCB2 crystal.

**Figure 5:**
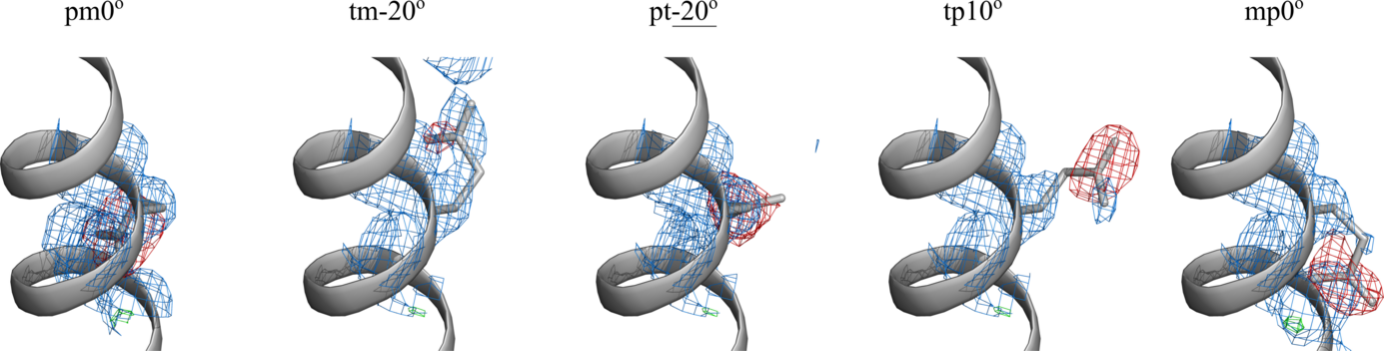
Side chain of residue Glu53 in the five specially prepared models of LCB2. The models represent the fully refined structure 8c3e, where the Glu53 side chain has been placed in pm0°, tm-20°, pt-20°, tp10° and mp0° conformations (as indicated in the plot). Electron density maps 2*mF*_o_ − *DF*_c_ are plotted at the level of 1σ (blue mesh) and the differential maps *mF*_o_ − *DF*_c_ are plotted at the level of 3σ (green/red mesh). The tm-20° model can be further refined, improving the agreement with its associated electron density (not shown).

This kind of logic leads us to further suggest that LCB2 models with incorrect Glu53 side-chain conformations (*i.e.*the ones that correspond to unpopulated or minimally populated rotamers) should fail to produce a viable solution. To test this assumption, we started with the fully refined LCB2 structure (8c3e, descendent from the *AlphaFold3* model) and placed the Glu53 side chain into one of the following conformational states: pm0°, tm-20°, pt-20°, tp10° or mp0° (*i.e.* all possible rotameric states except those three that have been already sampled, *cf*. Fig. 4 ▸). The resulting models were further used to generate the electron density maps, as shown in Fig. 5 ▸.

**Figure 6:**
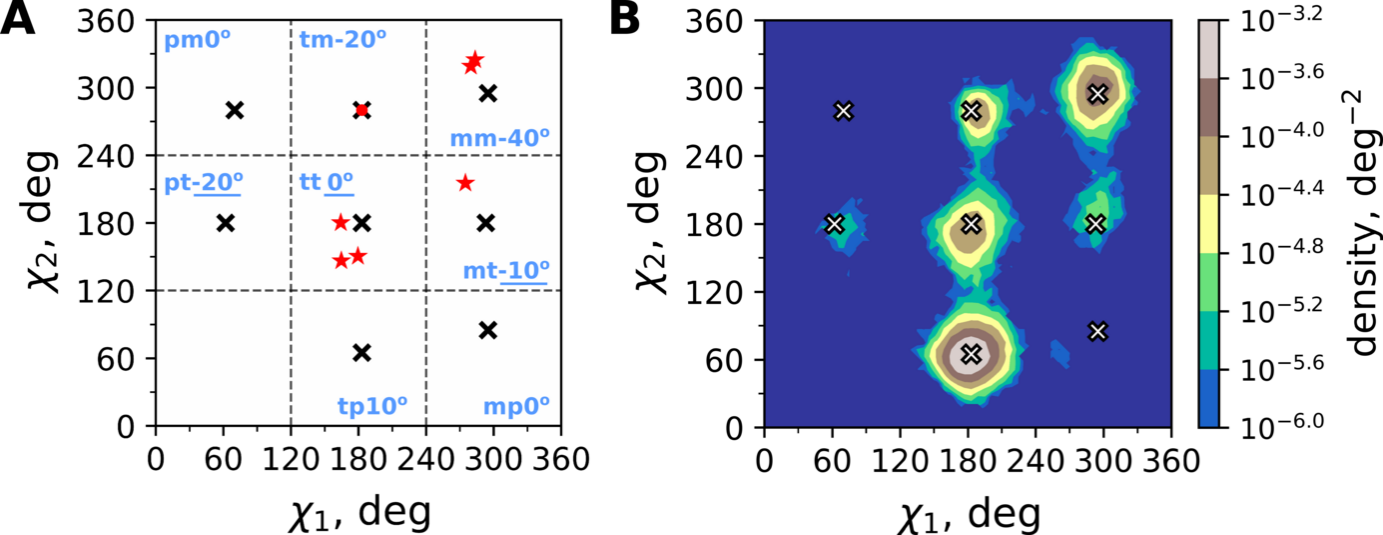
Conformational preferences of the Glu53 side chain in LCB2. (*a*) Crystallography-derived conformations from Fig. 4 ▸ (red asterisks) and Fig. 5 ▸ (tm-20°, red circle). Also indicated are canonical rotameric states of the Glu side chain (crosses) and their nomenclature (Lovell *et al.*, 2000 ▸). (*b*) Heat map showing (χ_1_, χ_2_) probability density distribution according to the MD simulation of the LCB2 crystal. The trajectory was started from the UC coordinates based on the structure 8c3e (descendent from the *AlphaFold3* model). For better visualization, the range of χ_1_ and χ_2_ is taken to be [0–360°]. The canonical rotameric states are indicated by crosses, as in panel (*a*).

The inspection of Fig. 5 ▸ shows that one of the models, tm-20°, is more-or-less consistent with the electron density, whereas the remaining four, pm0°, pt-20°, tp10° and mp0°, clearly fail the test as shown by negative density (red mesh). This outcome is in agreement with our interpretation, *i.e.* those models that match the actual side-chain conformations are supported by the electron density analyses, while other models are inconsistent with their associated electron density maps and hence can be discarded. A summary of the crystallographic results is presented in Fig. 6 ▸(*a*). In this plot, the six electron-density-supported conformations from Fig. 4 ▸ are shown with red asterisks and, in addition, the electron-density-supported conformation tm-20° from Fig. 5 ▸ is shown with a red circle.

To obtain further insight into the conformational dynamics of Glu53, we turn to MD simulations. In short, we used the six LCB2 structures obtained in this study to prepare six distinct models of the crystal unit cell (UC). Each UC model contained multiple protein molecules as well as intracrystalline solvent, which is explicitly represented by the optimal point charge (OPC) water (Izadi *et al.*, 2014 ▸). For each UC model we then recorded a 1 µs MD trajectory using the *Amber 2020* program (Case *et al.*, 2020 ▸) with the ff19SB force field (Tian *et al.*, 2020 ▸). To emulate the crystal lattice, periodic boundary conditions were applied to the unit-cell faces. The trajectories were recorded at 300 K in the isothermal–isobaric (NPT) ensemble using the Bussi–Parrinello velocity rescaling thermostat (Bussi *et al.*, 2007 ▸) (see the supporting information for further details).

In analyzing the MD data, we focused specifically on the conformation of the Glu53 side chain. Initially, each UC model featured its own Glu53 conformation, as illustrated in Fig. 4 ▸. However, as MD simulation progressed we observed Glu53 jumping from one rotameric state to another, eventually sampling a multitude of different conformations. The resulting rotamer distributions can be conveniently represented in the form of (χ_1_, χ_2_) heat maps; the third angle in the glutamate side chain, χ_3_, is only minimally restrained (Lovell *et al.*, 2000 ▸). Since the Glu53 side chain is highly dynamic, all six MD trajectories lead to near-identical distributions, thus indicating good convergence of the simulations, see Fig. S6. One representative map is shown in Fig. 6 ▸(*b*) (corresponding to the simulation that was started from the crystal coordinates of 8c3e).

Let us now review the results in Fig. 6 ▸. According to the MD data, there are three rotameric states that are sparsely populated (at the level of less than 1%): pm0°, pt-20° and mp0°. Indeed, none of these three rotameric states has been detected crystallographically, *i.e.* via electron density analyses. On the other hand, there are five rotameric states that are appreciably populated: tm-20°, mm-40°, tt 0°, mt-10° and tp10°. Four of these five states have been detected crystallographically. The sole exception is tp10°, which deserves a separate comment. According to the MD data, the population of this state reaches 65%. Note, however, that structural statistics reported by Lovell *et al.* suggest otherwise: on average, this conformational state is populated at the level of 6%, while specifically in α-helices it is populated at the level of 10% (Lovell *et al.*, 2000 ▸). Therefore, one may assume that MD simulations exaggerate the population of the tp10° state, which would explain why this conformation is not registered in our crystallographic study.

Moving beyond the Glu53 side chain, we assume that more generally multiple LCB2 structures obtained in this study can be viewed as an ensemble that is representative of the protein’s conformational dynamics. If so, then this six-member ensemble should give rise to lower values of *R*_work_/*R*_free_. Indeed, using the ensemble model comprising six LCB2 structures we arrive at the *R* factors of 0.187/0.220. This is significantly better than any of the structures taken alone; in fact, the best result from the individual structure is a good deal worse at 0.208/0.240 (see Table 2 ▸). Thus, we conclude that the collection of LCB2 structures obtained from the multi-start structure determination procedure as implemented in this work offers a useful (and in certain ways unique) representation of protein dynamics.

## 4. Concluding remarks

**Figure 7:**
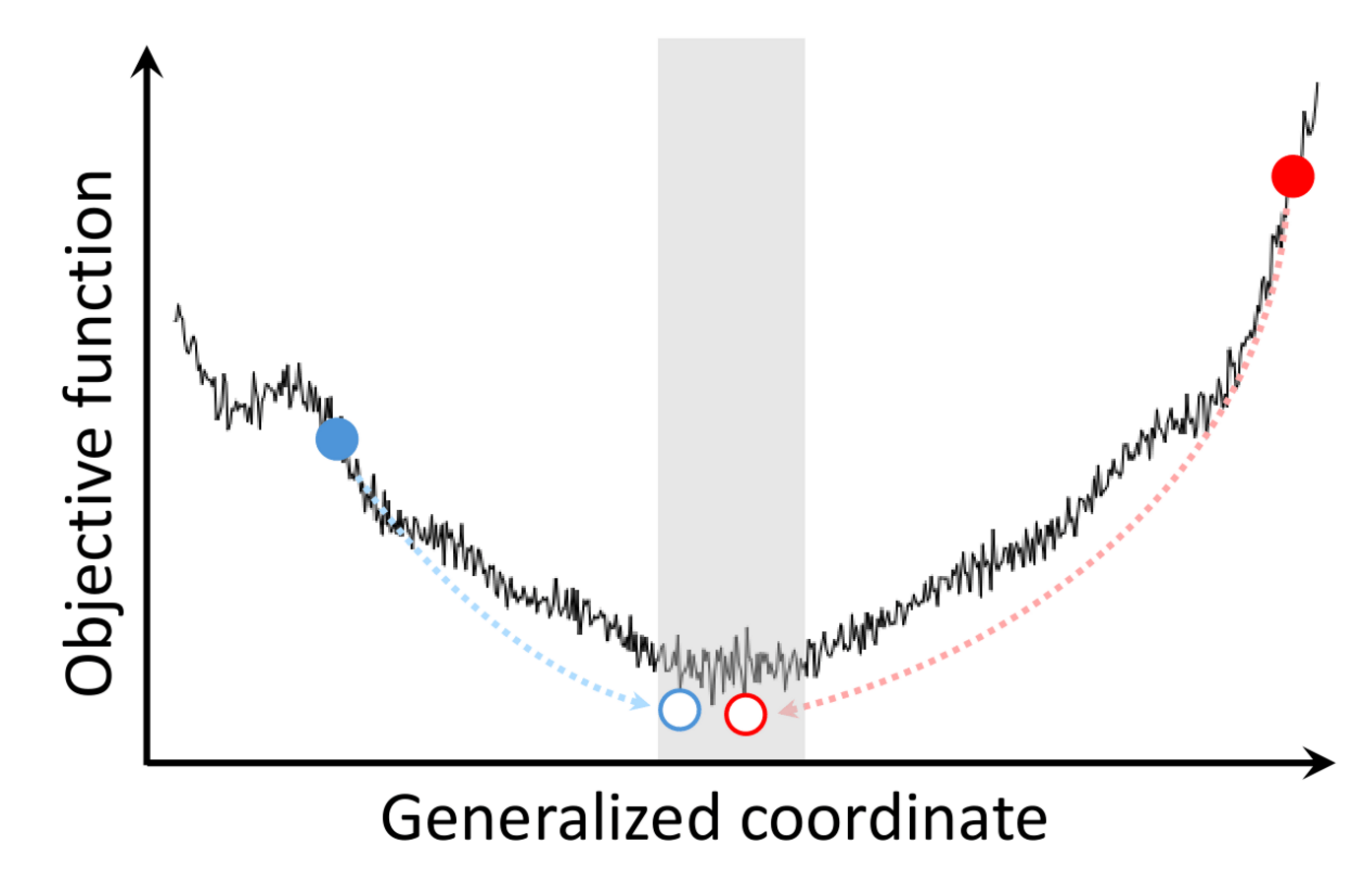
Protein structure calculation leads to a range of very similar, but distinct, structures, corresponding to the (roughly equiprobable) local minima in the objective function. Initial MR models and final (fully refined) structures are represented by solid and open circles, respectively.

Fig. 7 ▸ illustrates, in a schematic form, the process of solving a protein’s crystallographic structure. The objective function in the plot is the measure of agreement between the experimental and calculated structure factors. In practice, this is usually the maximum likelihood function (Murshudov *et al.*, 1997 ▸); alternatively, for the purpose of this discussion, it can be interpreted as the residual sum of squares or, otherwise, as the *R* factor. The generalized coordinate in the plot represents the complete set of atomic coordinates that defines the protein structure.

Solving the structure or, in a more narrow sense, refining the structure entails minimization of the objective function over the space of atomic coordinates. The process begins with an imprecise initial model (solid circle) and ends with a highly accurate structure (open circle). Up to a certain point, the minimization lowers the value of the objective function, until finally it arrives at the bottom of the well, where the algorithm settles in one of the multiple local minima (within the gray band in Fig. 7 ▸).

The multiple solutions are nearly identical, yet they differ from each other in certain subtle ways (*e.g.* with regard to conformations of certain side chains). Following DePristo, we argue that these solutions are equally valid (DePristo *et al.*, 2004 ▸). Small variations in *R*_free_ or other validation metrics are inconsequential and do not allow one to select the ‘best’ structure (*cf*. Table 2 ▸).

The multiplicity of the closely related solutions (numerous local minima within the gray band in the plot) stems from two factors: (1) conformational variability of the protein structure, *i.e.* local dynamics, and (2) uncertainty that is present in the diffraction data, *i.e.* limited resolution (Terwilliger *et al.*, 2007 ▸). The latter is itself mainly a consequence of dynamics, but also includes contributions from crystal defects, occasional radiation damage, finite accuracy of the X-ray detection system, approximate nature of the bulk solvent model *etc*. (Levin *et al.*, 2007 ▸; Holton *et al.*, 2014 ▸; Ma *et al.*, 2015 ▸).

It has long been recognized that using ensemble models instead of single-conformer models leads to somewhat better structural statistics. Broadly speaking, ensemble models can be generated by means of multi-copy optimization (Pellegrini *et al.*, 1997 ▸) or otherwise multi-start optimization (Rice *et al.*, 1998 ▸). The former approach involves simultaneous refinement of multiple protein copies, while the latter involves multiple (partially independent) refinement runs. It is believed that at higher crystallographic resolution ensemble models capture some of the actual conformational variability that is present in protein crystals and thus improve *R*_free_ and other measures of structural quality. At lower resolution, ensemble models still produce slightly better *R*_free_ values; in this case, the improvement is likely due to cancelation of small random errors associated with the individual solutions (similar to a standard procedure whereby the readouts from several repeat measurements are averaged to obtain a more accurate result).

The treatment described in this paper can be considered an example of multi-start refinement, where six final structures derived from different MR models are grouped together to form a small multiconformer ensemble. Unlike most implementations of ensemble refinement that were fully automated, we solved the structures using a traditional (labor-intensive) approach involving manual manipulations in *Coot*. In doing so, we observed an interesting effect whereby several dynamic side chains produced a different electron density footprint depending on the prior model. Apparently, when we use a (highly refined) model where the side-chain conformation corresponds to a rotameric state that is significantly populated in the actual crystal, we thereby elicit the electron density for this particular side-chain conformation. Conversely, when we use a model with an irrelevant (low-population) side-chain conformation, it fails to produce the matching electron density.

Strictly speaking, this behavior constitutes a case of model bias, *i.e.* certain details of the determined structure prove to be model dependent. We argue, however, that in the context of our study model bias plays a benign role, allowing us to characterize the rotameric distribution for the side chain at hand. Note that this would not be possible if we were to use standard tools for modeling of protein dynamics, such as alternative conformations.

One may wonder if the same result can be achieved by using a number of distinct structures derived from a single MR model (relying on the element of randomness which is present in both *Coot* manipulations and *Phenix* random-seeded refinement). To test this conjecture, we repeated the process of structure determination beginning from the *AlphaFold2*-predicted MR model. Indeed, we found several side chains adopting different conformations in the two *AlphaFold2*-derived solutions. As before, when considering individual structures, the electron density did not show any evidence of conformational dynamics at these sites. It is only through the comparison of the two structures that the conformational disorder became directly visible.

To further test the validity of rotameric distributions obtained for Glu53 and other dynamic side chains, we plan to use computer experiments (Levin *et al.*, 2007 ▸; Terwilliger *et al.*, 2007 ▸; van den Bedem *et al.*, 2009 ▸; Liu *et al.*, 2023 ▸). Specifically, we intend to simulate the diffraction data based on an MD trajectory of the LCB2 crystal and then use the simulated data to solve the protein structure using the same multi-start approach as described above. Comparison between the so-obtained multiconformer ensemble and the underlying MD model should directly confirm the interpretation presented in this work.

In summary, we have used six different computer-generated MR models to obtain a multiconformer ensemble for the small antiviral protein LCB2. While *R*_work_/*R*_free_ of the individual structures is at best 0.208/0.240, the six-member ensemble yields significantly improved values, 0.187/0.220. The procedure described in this paper potentially offers a new way to probe side-chain dynamics beyond the conventional electron density inspection. The proposed modeling scheme should add to the arsenal of methods that have been developed to glean information on protein dynamics from X-ray crystallography data.

## 5. Related literature

The following references are cited in the supporting information: Arbeitman *et al.* (2020 ▸); Atakisi *et al.* (2018 ▸); Berendsen *et al.* (1984 ▸); Dunlop *et al.* (2005 ▸); Essmann *et al.* (1995 ▸); Halle (2004 ▸); Hopkins *et al.* (2015 ▸); Jurrus *et al.* (2018 ▸); Kurauskas *et al.* (2017 ▸); Lebedenko *et al.* (2024 ▸); Poon (2010 ▸); Vonrhein *et al.* (2011 ▸); Webb & Sali (2016 ▸).

## Supplementary Material

Supporting information. DOI: 10.1107/S2052252525005123/lz5075sup1.pdf

PDB reference: engineered mini-protein LCB2 (blocking ligand of SARS-CoV-2 spike protein), 8c3e

All six starting models, final structures and final electron density maps: https://doi.org/10.5281/zenodo.15337018

## Figures and Tables

**Table 1:** Potential MR models for the mini-protein LCB2 obtained from different structure predictors and their *Phaser*-determined LLG and TFZ scores The PDB was accessed on 30 April 2025.

Model	*Phaser* LLG score	*Phaser* TFZ score	No. of MR models in the PDB†	First use as an MR model in the PDB‡
*AlphaFold3*	213	16	2142§	2021
*AlphaFold2*	208	17		
*MultiFOLD*	176	15	0	—
*Rosetta*	105	12	71	2007
*RoseTTAFold*	94	11	46	2021
*trRosetta*	87	10	2	2021
				
*QUARK*	88	5	2	2018
*Phyre2*	82	5	32¶	2005
*I-TASSER*	57	5	19	2011
*SWISS-MODEL*	25	5	170	1999

† The number of identifiable PDB structures solved by using MR models from a given predictor.

‡ The release date of the earliest such PDB structure.

§ Probably includes a small number of models from the older program *AlphaFold*.

¶ Probably includes a small number of models from the older program *Phyre*.

**Table 2:** Refinement statistics for LCB2 structures solved using different computer-predicted MR models

	Predictor					
	*AlphaFold3*	*AlphaFold2*	*MultiFOLD*	*Rosetta*	*RoseTTAFold*	*trRosetta*
*R*_work_/*R*_free_	0.209/0.249	0.212/0.240	0.214/0.252	0.214/0.245	0.208/0.240	0.211/0.254
*MolProbity* score (percentile)	0.50 (100)	0.50 (100)	0.98 (100)	0.98 (100)	0.80 (100)	0.80 (100)
						
Ramachandran						
Favored (%)	98	100	100	98	100	98
Allowed (%)	2	—	—	2	—	2
Outliers (%)	—	—	—	—	—	—
						
Side chains						
Rotameric (%)	100	100	100	100	100	100
Outliers (%)	—	—	—	—	—	—
						
Clash score	0.00	0.00	2.06	2.06	1.02	1.02
						
RMS deviations						
Bond lengths (Å)	0.002	0.002	0.002	0.002	0.002	0.002
Angles (°)	0.36	0.38	0.40	0.40	0.38	0.36
						
No. of atoms						
Protein	469	469	469	469	469	469
Water	18	18	18	20	18	24
Co-solvent (glycerol)	24	6	12	12	18	18
						
Average *B* (Å^2^)	14	13	12	13	12	12
						
PDB deposition	8c3e	—	—	—	—	—

## Data Availability

The LCB2 structure obtained from the *AlphaFold3*-generated model has been deposited in Protein Data Bank (PDB ID 8c3e). All six starting models, final structures and final electron density maps are available from the Zenodo repository, https://doi.org/10.5281/zenodo.15337018.
