# Supplementary material for: Using multiple computer-predicted structures as molecular replacement models: application to the antiviral mini-protein LCB2
Source: IUCrJ. 2025 Jun 23;12(Pt 4):488–501. doi: 10.1107/S2052252525005123 (PMC12224078; doi:10.1107/S2052252525005123)
Supplement: Supplementary file 1 [file m-12-00488-sup1.pdf]

IUCrJ

Volume 12 (2025)

Supporting information for article:

Using multiple computer-predicted structures as molecular replacement models: application to the antiviral mini-protein LCB2

Svetlana A. Korban, Oleg Mikhailovskii, Vladislav V. Gurzhiy, Ivan S. Podkorytov and Nikolai R. Skrynnikov

SUPPORTING INFORMATION

Using multiple computer-predicted structures as molecular replacement models: application to antiviral mini-protein LCB2

Svetlana A. Korban,[1,2] Oleg Mikhailovskii,[3] Vladislav V. Gurzhiy,[4] Ivan S. Podkorytov,[1] Nikolai R. Skrynnikov[1,5]

[1] *Laboratory of Biomolecular NMR, St. Petersburg State University, St. Petersburg 199034, Russia*
[2] *NRC «Kurchatov Institute» – PNPI, Gatchina 188300, Russia*
[3] *Independent researcher, Utrecht, the Netherlands*
[4] *Crystallography Department, Institute of Earth Sciences, St. Petersburg State University, St. Petersburg 199034, Russia*
[5] *Department of Chemistry, Purdue University, West Lafayette, IN 47907, USA*

E-mail: n.skrynnikov@spbu.ru

1. Protein expression and purification

cDNA of LCB2, including additionally an N-terminal His_6-tag and thrombin cleavage site, has been cloned into pET-15b vector and used to transform *E. coli* Rosetta DE3 cells. The protein was expressed in a standard manner (induction with 1 mM IPTG at OD_{600} = 0.6-0.8 followed by 16-18 h incubation at 37 °C). Thereafter the cells were lysed using the cryogenic grinder and the protein was purified by means of metal affinity chromatography (HisTrap HP, Thermo Fisher Scientific). The protein was subsequently treated with thrombin to remove the histidine tag (16-18 h at room temperature) and additionally purified by means of size-exclusion chromatography (Sephacryl S-200 HR, Merck). The sample was obtained with the following amino-acid sequence, GSSDDEDSVRYLLYMAELRYEQGNPEKAKKILEMAEFIAKRNNNEELERLVREVKKRL, which corresponds to the original LCB2 sequence (Cao *et al.*, 2020) plus the N-terminal GS residues left over from the thrombin cleavage site. The preparation was validated by means of MALDI-TOF (expected mass 6916.78 Da, the obtained mass 6916.53 Da). We have also prepared ^{15}N-labeled sample of LCB2 and recorded $^{1}H^{N}$,^{15}N-HSQC spectrum of LCB2 (0.3 mM protein in 20 mM phosphate, 150 mM NaCl at pH 7.4). The spectrum indicates that the protein is well folded under these (physiologically relevant) conditions. At the same time, some of the peaks are significantly broadened; furthermore, the number of observable peaks is smaller than expected, suggestive of weakly specific self-association of LCB2 under the experimental conditions.

2. Isothermal titration calorimetry

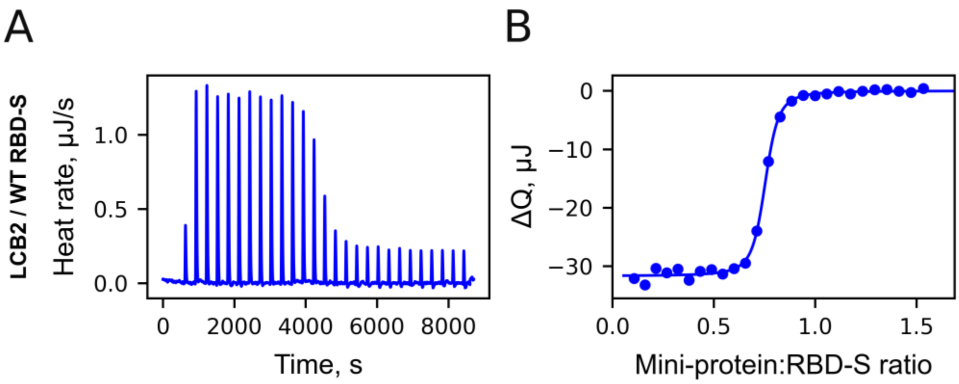

Figure S1. (A) ITC thermogram for binding of the mini-protein LCB2 to the WT RBD-S of SARS-CoV-2. (B) ITC data after baseline correction, peak integration and blank subtraction. The data have been fitted using the python script written in-house.

The functional fitness of the obtained LCB2 sample was tested by means of the isothermal titration calorimetry (ITC) test to confirm LCB2 binding to the receptor-binding domain of the spike protein (RBD-S) from SARS-CoV-2. For this purpose, the recombinant RBD-S (historic Wuhan strain) was expressed in *P. pastoris* X-33 cells and purified chromatographically as described previously (Arbeitman *et al.*, 2020). The degassed solution of RBD-S with the concentrations 5 µM was titrated with 50 µM stock solution of LCB2 using Nano ITC 2G microcalorimeter (TA Instruments). The injection volume was 5.13 µL and the interval between the injections was 300 s. After baseline correction and peak integration, the data were fitted using a standard single-site (non-competitive) binding model (Poon, 2010) (see Fig. S1). The experiment yields stoichiometry 1.01, binding enthalpy ΔH = -81.6 ± 1.3 kJ/mol and dissociation constant K_d = 8.6 ± 2.1 nM.

3. Protein crystallization, data collection and processing

Purified LCB2 protein was crystallized using the hanging-drop vapor-diffusion technique. Crystals were grown at 18 °C in hanging drops consisting of equal volumes of protein solution and precipitant (50 мM ammonium sulphate, 0.1 M sodium acetate at pH 4.81, 5% w/v PEG 4000) for at least five days. The resulting needle crystals were collected, soaked in mother liquor supplemented with 20% glycerol as cryoprotectant and then flash-frozen in liquid nitrogen for further transportation and data collection (see Tab. S1 for additional details). Diffraction data were collected at 100 K on a XtaLAB Synergy-S diffractometer equipped with PhotonJet-S Cu microfocus X-ray source and HyPix-6000HE detector (Rigaku Oxford Diffraction, Oxford, UK) at the Center for XRD studies at SPbU. Indexing, integration and scaling were performed using autoPROC 1.0.5 (Vonrhein *et al.*, 2011). The summary of diffraction data is given in Tab. S2. The electron density maps obtained in the process of structure determination contain some low-amplitude noise-like features clustered near the axis of the solvent channel. Standard validation tests in Coot searching for unmodeled blobs in $2mF_o - DF_c$ and $mF_o - DF_c$ maps with cutoff levels set to 1.4σ and 3σ respectively do not pick up on these features, thus confirming their noise-like character.

Method	Hanging-drop vapour diffusion
Temperature	18 °C
Protein concentration	10 mg/mL
Buffer composition of protein solution	20 mM HEPES pH 7.4, 150 mM NaCl, 0.01% NaN_3
Composition of reservoir solution	0.1 M Sodium acetate pH 4.8, 50 mM ammonium sulfate, 5% PEG 4000
Drop volume and mixing ratio	2.0 µl, 1:1 (protein:reservoir)

Table S1. Crystallization parameters.

Space group	$P3_121$
a, *b*, *c* (Å)	57.82, 57.82, 41.54
α, β, γ (°)	90, 90, 120
Resolution range (Å)	50.07 - 2.10 (2.14 - 2.10)
No. of unique reflections	4881 (234)
Completeness (%)	99.9 (100)
Multiplicity	10.2 (10.7)
$\langle I/\sigma(I)\rangle$	16 (4)
R_{merge}	0.156 (0.610)
R_{meas}	0.164 (0.641)
Half-set correlation $CC_{1/2}$	0.997 (0.859)
Overall *B* factor from Wilson plot (Å^2)	11.9
L-test for twinning[1]	$\langle\lvert L\rvert\rangle$ = 0.40, $\langle L^2\rangle$= 0.23
Estimated twinning fraction	0.447 for -h,-k,l

Table S2. Diffraction data statistics. Values in parentheses are for the outer shell.

[1] Theoretical values of $\langle\lvert L\rvert\rangle$, $\langle L^2\rangle$ for acentric reflections are 0.5, 0.333 for untwinned datasets and 0.375, 0.2 for perfectly twinned datasets. To avoid potential bias in R_{free} due to twinning, the test reflections were selected in thin resolution shells.

4. Additional information on MR models

AlphaFold3	https://alphafoldserver.com/	used in this study, current
AlphaFold2	https://colab.research.google.com/github/deepmind/alphafold/blob/4d83e3fc0883011e3d597eb6d33e532267754708/notebooks/AlphaFold.ipynb	used in this study
	https://colab.research.google.com/github/sokrypton/ColabFold/blob/main/AlphaFold2.ipynb	current
MultiFOLD	https://www.reading.ac.uk/bioinf/MultiFOLD/	used in this study, current
Rosetta	http://files.ipd.uw.edu/pub/SARS-CoV-2_binder_2020/scripts_models.zip	used in this study, current
RoseTTAFold	https://robetta.bakerlab.org/	used in this study, current
trRosetta	https://yanglab.nankai.edu.cn/trRosetta/	used in this study
	https://yanglab.qd.sdu.edu.cn/trRosetta/	current

Table S3. Sources of MR models used in this study: structure prediction servers and file archive.

Model	LLG/TFZ		
	B-factors from ASA	B-factors from pLDDT or RMSD	B-factors constant
AlphaFold3_model0	176/14	179/14	138/12
AlphaFold3_model1	195/16	201/16	146/13.5
AlphaFold3_model2	177/14	186/15	155/13
AlphaFold3_model3	157/13.5	170/14	125/12
AlphaFold3_model4	202/16	**213/16**	159/14
AlphaFold2_v2.3.2	**208/17**	188/16	167/15
MultiFOLD_model1	155/15	149/15	132/14
MultiFOLD_model2	157/15	153/15	136/14
MultiFOLD_model3	135/14	128/12	111/12
MultiFOLD_model4	173/16	**176/15**	148/14
MultiFOLD_model5	169/14	158/14	147/14
Rosetta	**105/12**	-	91/11
RoseTTAFold_model1	84/10	91/11	77/9
RoseTTAFold_model2	74/9	83/10	67/9
RoseTTAFold_model3	85/10	**94/11**	82/10

RoseTTAFold_model4	80/9.5	90/10	78/9
RoseTTAFold_model5	58/8	85/10	54/7
trRosetta_model1	84/10	68/9	59/8
trRosetta_model2	82/9	68/8	59/8
trRosetta_model3	**87/10**	68/9	65/8.5
trRosetta_model4	87/9	74/9	66/8.5
trRosetta_model5	65/8	70/9	60/8
QUARK_model1	21/5	46/6	16/5
QUARK_model2	27/5	71/7	25/5
QUARK_model3	28/5	43/6	25/6
QUARK_model4	45/8	**88/5**	24/5
QUARK_model5	54/9	60/5	32/6
Phyre2_model1	30/6	-	26/6
Phyre2_model2	45/6	-	43/6
Phyre2_model3	20/5	-	20/5
Phyre2_model4	**82/5**	-	40/6
Phyre2_model5	47/6	-	41/6
I-TASSER_model1	22/5	**57/5**	23/6
I-TASSER_model2	27/6	-	21/5
I-TASSER_model3	40/7	-	39/7
SWISS-MODEL	24/5	-	25/5

Table S4. Assignment of B-factors to potential MR models. Summary of Phaser-determined LLG/TFZ scores for predicted LCB2 models with different B-factor assignment strategies: based on accessible surface area (*phenix.sculptor*), based on pLDDT scores or RMSD values (*phenix.process_predicted_model*), or set to a constant value (e.g. 10 or 20). The productive models are typeset in color, with the best model in each group (as judged by the LLG score) typeset in bold. Further details regarding the productive models can be found in the *Materials and methods* section of the article.

5. Alternative metrics to compare structural models

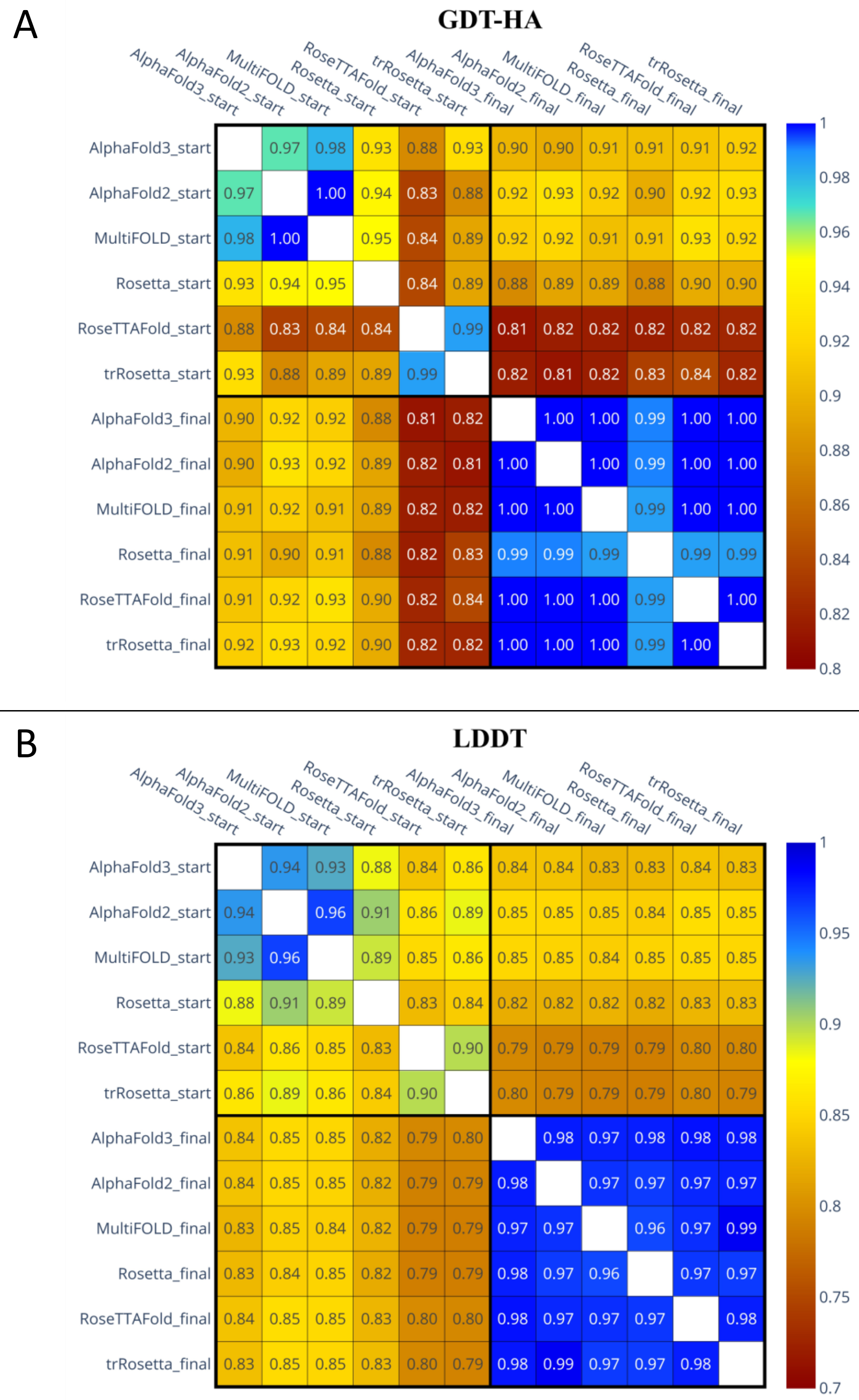

Figure S2. Pairwise deviations between six computer-predicted MR models and six resultant fully refined structures of LCB2 expressed in a form of (A) GDT-HA and (B) LDDT (Olechnovič *et al.*, 2019). Both metrics have been calculated for all non-hydrogen atoms in the "consensus set" (shared by all models/structures, see *Materials and methods*).

6. Principal component analysis

Prior to the analyses, all sets of coordinates (six MR models and six fully refined structures) are superimposed via all non-hydrogen atoms in the consensus set. The resulting structures are then treated as row vectors $\vec{R}_l$. These vectors are comprised of all non-hydrogen atoms in the consensus set (vector length 435·3=1305). As a first step, the mean is subtracted from the data:

$$\vec{r}_l = \vec{R}_l - \frac{1}{12}\sum_{k=1}^{12}\vec{R}_k \quad \text{(S1).}$$

The resulting row vectors $\vec{r}_l$ are then arranged into matrix $\hat{r}$ which has the dimensions 12×1305.

Following the standard approach in the principal component analysis (PCA), we search for a unit row vector $\vec{p}$ that maximizes the function

$$S(\vec{p}) = \sum_{l=1}^{12}(\vec{r}_l \cdot \vec{p})^2 \quad \text{(S2),}$$

i.e. maximizes the spread in the projections of the 12 vectors $\vec{r}_l$ onto the axis defined by $\vec{p}$. The function $S(\vec{p})$ can be further expressed through the covariance matrix $\hat{K} = \hat{r}^T\hat{r}$:

$$S(\vec{p}) = \vec{p}\hat{K}\vec{p}^T \quad \text{(S3).}$$

It is then straightforward to show that the maximum of $S(\vec{p})$ is achieved for $\vec{p} = \vec{p}_1$, where $\vec{p}_1$ is the unit left eigenvector of the covariance matrix $\hat{K}$ corresponding to the largest eigenvalue λ_1 (Jolliffe, 2002).

Next, searching for a maximum of $S(\vec{p})$ among the vectors $\vec{p}$ that are orthogonal to $\vec{p}_1$ leads to $\vec{p} = \vec{p}_2$, where $\vec{p}_2$ is the unit left eigenvector of $\hat{K}$ corresponding to the second largest eigenvalue λ_2. Similarly, searching for a maximum of $S(\vec{p})$ among the vectors $\vec{p}$ that are orthogonal to both $\vec{p}_1$ and $\vec{p}_2$ results in $\vec{p} = \vec{p}_3$, where $\vec{p}_3$ is the unit left eigenvector of $\hat{K}$ corresponding to the third largest eigenvalue λ_3, etc.

In this standard manner we obtain eleven orthogonal unit vectors $\vec{p}_k$ corresponding to non-zero eigenvalues λ_k of the covariance matrix $\hat{K}$. These vectors form a basis that spans the space of the twelve original vectors $\vec{r}_l$ (only eleven out of twelve are linearly independent because they add up to zero, see Eq. (S1)). For all of the vectors $\vec{r}_l$ we define their projections on $\vec{p}_1$ as $\mathrm{PC1}_l = (\vec{r}_l \cdot \vec{p}_1)$, their projections on $\vec{p}_2$ as $\mathrm{PC2}_l = (\vec{r}_l \cdot \vec{p}_2)$ and so forth. Traditionally, the investigators pay attention to PC1, PC2 and sometimes PC3, since those components highlight the differences between the investigated objects (in our case, structural models of LCB2).

To perform numeric calculations, we used the Matlab function pca, applying it to our data matrix $\hat{r}$:

$$[c, s, \dots] = pca(\hat{r}) \quad \text{(S4).}$$

The output matrix c is comprised of vectors $\vec{p}_k$ arranged as columns (matrix dimensions 1305×11). The output matrix s stores the principal-component representation of the vectors $\vec{r}_l$, $s_{lk} = (\vec{r}_l \cdot \vec{p}_k)$ (matrix dimensions 12×11).

As a part of our analyses, we also visualized the structural differences associated only with PC1 or PC2 components for a given pair of models (e.g. initial AlphaFold3 model and the corresponding final structure). For example, consider the *i*-th model described by a set of principal components

$\{\mathrm{PC1}_i, \mathrm{PC2}_i, \mathrm{PC3}_i, \dots, \mathrm{PC11}_i\}$ and the *j*-th model described by $\{\mathrm{PC1}_j, \mathrm{PC2}_j, \mathrm{PC3}_j, \dots, \mathrm{PC11}_j\}$. In order to focus on structural differences associated with the first component, we generate a pair of pseudo-models, where the individual components PC1 are retained, whereas all other components are averaged, $\{\mathrm{PC1}_i, (\mathrm{PC2}_i + \mathrm{PC2}_j)/2, (\mathrm{PC3}_i + \mathrm{PC3}_j)/2, \dots, (\mathrm{PC11}_i + \mathrm{PC11}_j)/2\}$ and $\{\mathrm{PC1}_j, (\mathrm{PC2}_i + \mathrm{PC2}_j)/2, (\mathrm{PC3}_i + \mathrm{PC3}_j)/2, \dots, (\mathrm{PC11}_i + \mathrm{PC11}_j)/2\}$. These pseudo-models can then be easily transformed into vectors in the $\vec{r}$ space, converted into PDB-style files and visualized using any appropriate viewer program such as PyMOL.

7. Side-chain dynamics

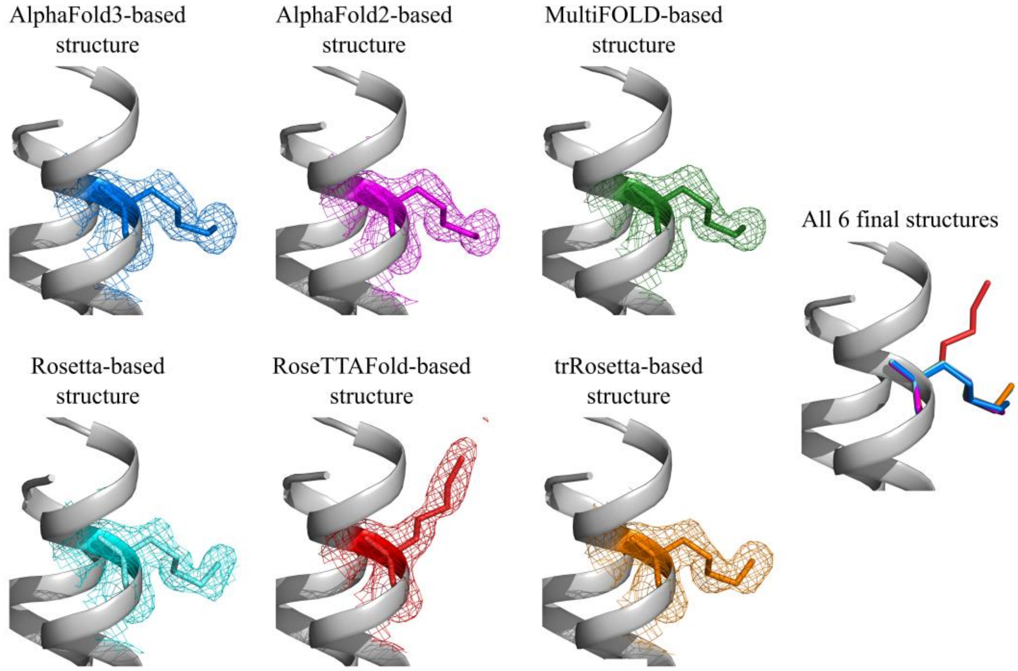

Figure S3. Side chain of residue K29 in the six final structures of LCB2 and the respective electron density maps $2mF_o - DF_c$ plotted at the level of 1σ. Color scheme is the same as in Fig. 4.

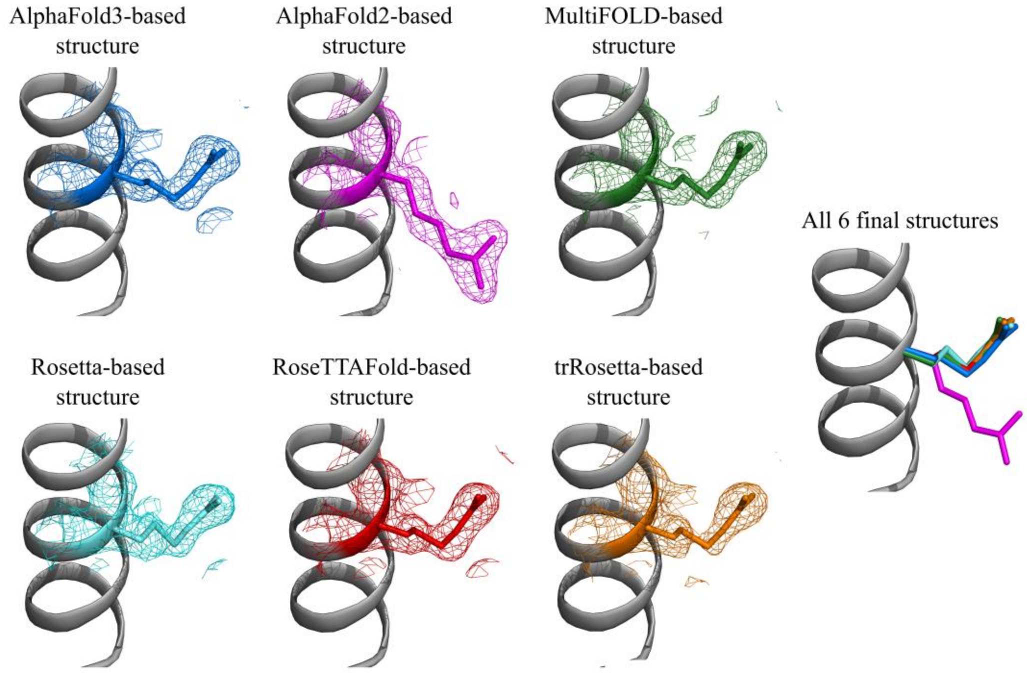

Figure S4. Side chain of residue R52 in the six final structures of LCB2 and the respective electron density maps $2mF_o - DF_c$ plotted at the level of 1σ. Color scheme is the same as in Fig. 4.

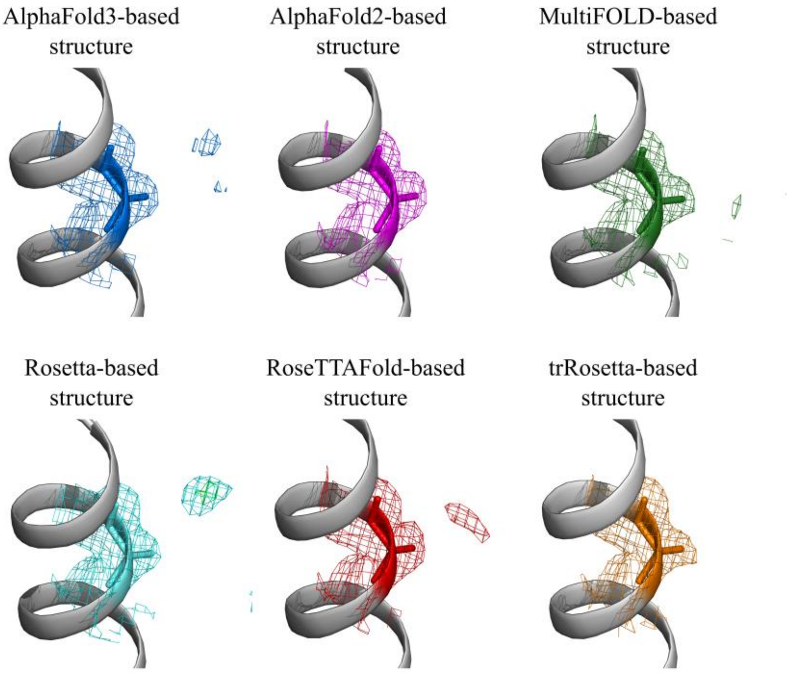

Figure S5. OMIT maps for residue E53. To prepare this plot, we took six final LCB2 structures, truncated their E53 side chains beyond Cβ, and used the resulting constructs to generate $2mF_o - DF_c$ maps. The maps are plotted at the level 1σ using the same coloring scheme as in Fig. 4.

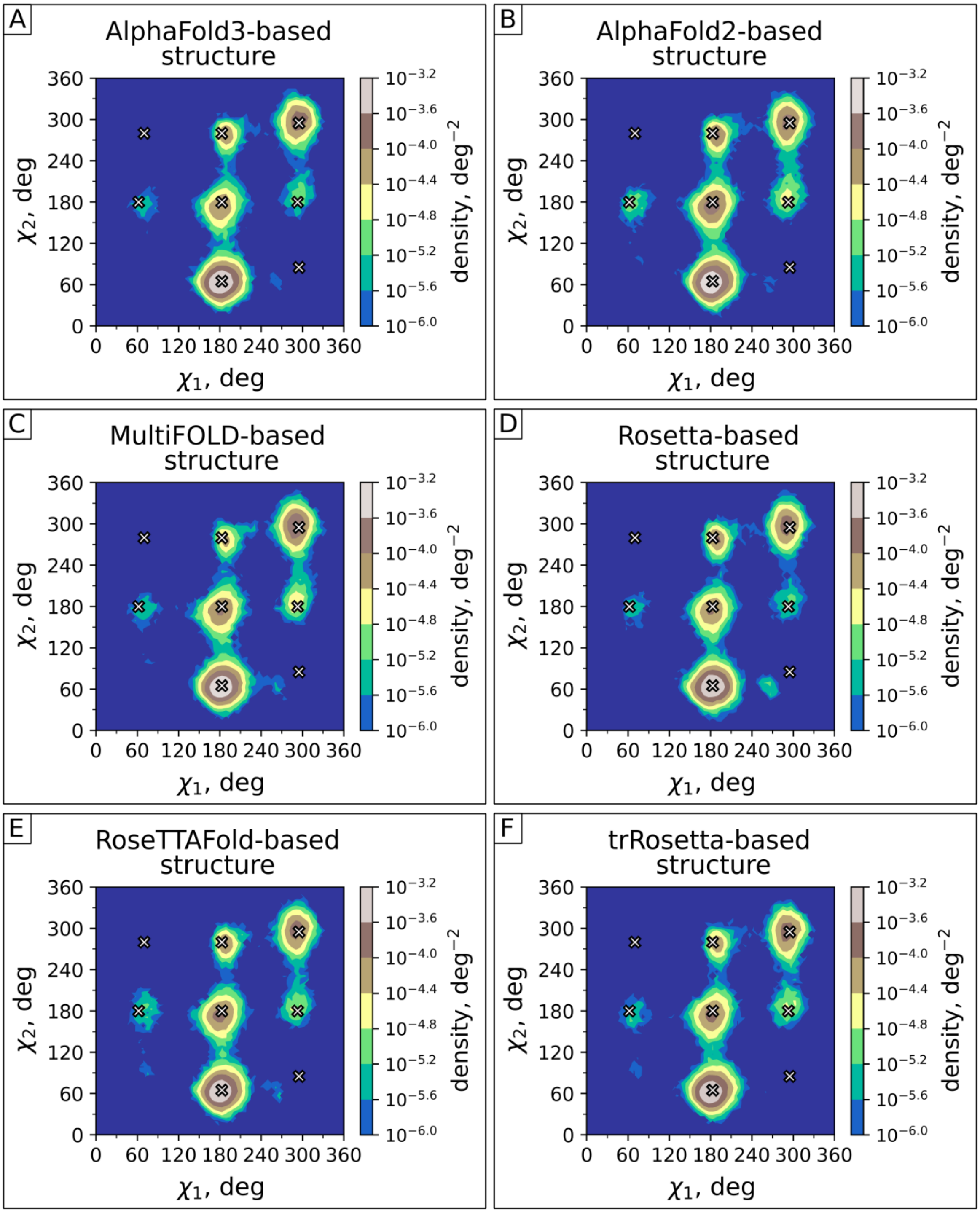

Figure S6. Heat maps showing (χ_1, χ_2) probability density distribution for E53 side chain as obtained from our MD simulations of the LCB2 crystals. Six 1-µs-long trajectories have been started from the UC models involving different LCB2 structures, as indicated above the panels (further details on MD simulations are provided in the SI section 8, see below). Panel A from this plot is also reproduced in Fig. 6(*b*).

8. MD simulations

As a starting point, we have used six fully refined LCB2 structures obtained from different computer-predicted MR models. Those residues that are missing at the N-terminus have been rebuilt using the program Modeller (Webb & Sali, 2016). Beginning with these structures we assembled six different unit-cell (UC) models. For this purpose we used the procedure *UnitCell* in the package Amber 20. The parameters of the unit cell are as listed in Tab. S2. To determine the titration states of the ionizable side chains and protonate protein molecules, we have used the program PDB2PQR (Jurrus *et al.*, 2018). The effective pH was assumed to be 4.8, same as in the crystallization buffer. The system was neutralized by adding Cl^- ions. OPC water (as well as chlorine ions) were added to the UC model by means of the *AddToBox* facility in Amber 20. The number of water molecules in the UC box has been further adjusted such as to ensure that the box maintains its original size during the course of MD modeling (Kurauskas *et al.*, 2017). Additives such as polyethylene glycol or glycerol have not been added to the model since it is unclear to what degree they are partitioned into the crystals.

Prior to the production run, the UC models have been relaxed. At first, water coordinates were optimized while protein atoms were fixed; then all restraints were lifted and the entire model was energy-minimized. After that the temperature of the system was raised from 0 to 300 K by running 200 ps constant-volume simulation with weak restraints applied to all protein atoms (10 kcal mol^{-1} $Å^{-2}$). Thereafter the production run was initiated. The first nanosecond in each trajectory was treated as equilibration period and subsequently discarded.

The simulations were run in the NPT ensemble with the pressure of 1 atm and the temperature of 300 K. The temperature was controlled by the Bussi-Parinello velocity rescaling thermostat (Bussi *et al.*, 2007) with 1 ps^{-1} coupling frequency. The pressure was maintained by Berendsen barostat (Berendsen *et al.*, 1984) with isotropic scaling and a pressure relaxation time of 2 ps. Long-range electrostatics were treated with the particle mesh Ewald method (Essmann *et al.*, 1995); non-bonded Coulomb and Lennard-Jones interactions were truncated at 9 Å. The bonds involving hydrogen atoms were constrained using SHAKE algorithm. The hydrogen mass repartition (HMR) scheme (Hopkins *et al.*, 2015) was applied to all components in the system, including water, thus facilitating the use of 4-fs integration timestep (Lebedenko *et al.*, 2024). The production rate using NVIDIA GeForce RTX2080 Ti cards was 500 ns per card per day.

The simulation temperature of 300 K has been chosen to obtain adequate sampling of E53 conformational dynamics and also because conventional force fields, such as Amber ff19SB, are best suited to model protein behavior at ambient conditions. While there are some differences in rotameric preferences of protein side chains between room temperature and 100 K (Dunlop *et al.*, 2005), these differences are rather limited; they are caused by complex factors such as remodeling of crystal contacts upon compaction of the crystal at cryogenic temperatures and the influence of cryoprotectants (Atakisi *et al.*, 2018). One should also bear in mind that rotameric distributions observed in crystallographic structures that have been solved at 100 K are actually representative of higher temperature, estimated to be near 200 K (Halle, 2004). Given the complexity of these effects, we reason that room-temperature simulations is the most practical option to identify a conformationally labile residue such as E53.

The MD trajectories were processed and torsional angles were extracted using python library, *pyxmolpp2*, written in-house by S.A. Izmailov (see https://github.com/bionmr-spbu/pyxmolpp2).

Supporting references

Arbeitman, C. R., Auge, G., Blaustein, M., Bredeston, L., Corapi, E. S., Craig, P. O., Cossio, L. A., Dain, L., D'Alessio, C., Elias, F., Fernandez, N. B., Gandola, Y. B., Gasulla, J., Gorojovsky, N., Gudesblat, G. E., Herrera, M. G., Ibanez, L. I., Idrovo, T., Rando, M. I., Kamenetzky, L., Nadra, A. D., Noseda, D. G., Pavan, C. H., Pavan, M. F., Pignataro, M. F., Roman, E., Ruberto, L. A. M., Rubinstein, N., Santos, J., Velazquez, F. & Zelada, A. M. (2020). *Sci. Rep.* **10**, 21779.

Atakisi, H., Moreau, D. W. & Thorne, R. E. (2018). *Acta Cryst. D* **74**, 264-278.

Berendsen, H. J. C., Postma, J. P. M., van Gunsteren, W. F., Dinola, A. & Haak, J. R. (1984). *J. Chem. Phys.* **81**, 3684-3690.

Bussi, G., Donadio, D. & Parrinello, M. (2007). *J. Chem. Phys.* **126**, 014101.

Cao, L., Goreshnik, I., Coventry, B., Case, J. B., Miller, L., Kozodoy, L., Chen, R. E., Carter, L., Walls, A. C., Park, Y.-J., Strauch, E.-M., Stewart, L., Diamond, M. S., Veesler, D. & Baker, D. (2020). *Science* **370**, 426-431.

Dunlop, K. V., Irvin, R. T. & Hazes, B. (2005). *Acta Cryst. D* **61**, 80-87.

Essmann, U., Perera, L., Berkowitz, M. L., Darden, T., Lee, H. & Pedersen, L. G. (1995). *J. Chem. Phys.* **103**, 8577-8593.

Halle, B. (2004). *Proc. Natl. Acad. Sci. USA* **101**, 4793-4798.

Hopkins, C. W., Le Grand, S., Walker, R. C. & Roitberg, A. E. (2015). *J. Chem. Theory Comput.* **11**, 1864-1874.

Jolliffe, I. T. (2002). *Principal Component Analysis*, 2-nd ed. New York: Springer.

Jurrus, E., Engel, D., Star, K., Monson, K., Brandi, J., Felberg, L. E., Brookes, D. H., Wilson, L., Chen, J., Liles, K., Chun, M., Li, P., Gohara, D. W., Dolinsky, T., Konecny, R., Koes, D. R., Nielsen, J. E., Head-Gordon, T., Geng, W., Krasny, R., Wei, G. W., Holst, M. J., McCammon, J. A. & Baker, N. A. (2018). *Protein Sci.* **27**, 112-128.

Kurauskas, V., Izmailov, S. A., Rogacheva, O. N., Hessel, A., Ayala, I., Woodhouse, J., Shilova, A., Xue, Y., Yuwen, T., Coquelle, N., Colletier, J. P., Skrynnikov, N. R. & Schanda, P. (2017). *Nat. Commun.* **8**, 145.

Lebedenko, O. O., Salikov, V. A., Izmailov, S. A., Podkorytov, I. S. & Skrynnikov, N. R. (2024). *Biophys. J.* **123**, 80-100.

Olechnovič, K., Monastyrskyy, B., Kryshtafovych, A. & Venclovas, Č. (2019). *Bioinformatics* **35**, 937-944.

Poon, G. M. K. (2010). *Anal. Biochem.* **400**, 229-236.

Vonrhein, C., Flensburg, C., Keller, P., Sharff, A., Smart, O., Paciorek, W., Womack, T. & Bricogne, G. (2011). *Acta Cryst. D* **67**, 293-302.

Webb, B. & Sali, A. (2016). *Curr. Protoc. Bioinform.* **54**, 5.6.1-5.6.37.
